# vaRHC: an R package for semi-automation of variant classification in hereditary cancer genes according to ACMG/AMP and gene-specific ClinGen guidelines

**DOI:** 10.1093/bioinformatics/btad128

**Published:** 2023-03-14

**Authors:** Elisabet Munté, Lidia Feliubadaló, Marta Pineda, Eva Tornero, Maribel Gonzalez, José Marcos Moreno-Cabrera, Carla Roca, Joan Bales Rubio, Laura Arnaldo, Gabriel Capellá, Jose Luis Mosquera, Conxi Lázaro

**Affiliations:** Hereditary Cancer Program, Program in Molecular Mechanisms and Experimental Therapy in Oncology (Oncobell), IDIBELL, Catalan Institute of Oncology, L’Hospitalet de Llobregat 08908, Spain; Hereditary Cancer Program, Program in Molecular Mechanisms and Experimental Therapy in Oncology (Oncobell), IDIBELL, Catalan Institute of Oncology, L’Hospitalet de Llobregat 08908, Spain; Centro de Investigación Biomédica en Red de Cáncer (CIBERONC), Madrid, Spain; Hereditary Cancer Program, Program in Molecular Mechanisms and Experimental Therapy in Oncology (Oncobell), IDIBELL, Catalan Institute of Oncology, L’Hospitalet de Llobregat 08908, Spain; Centro de Investigación Biomédica en Red de Cáncer (CIBERONC), Madrid, Spain; Hereditary Cancer Program, Program in Molecular Mechanisms and Experimental Therapy in Oncology (Oncobell), IDIBELL, Catalan Institute of Oncology, L’Hospitalet de Llobregat 08908, Spain; Hereditary Cancer Program, Program in Molecular Mechanisms and Experimental Therapy in Oncology (Oncobell), IDIBELL, Catalan Institute of Oncology, L’Hospitalet de Llobregat 08908, Spain; Hereditary Cancer Program, Program in Molecular Mechanisms and Experimental Therapy in Oncology (Oncobell), IDIBELL, Catalan Institute of Oncology, L’Hospitalet de Llobregat 08908, Spain; Hereditary Cancer Program, Program in Molecular Mechanisms and Experimental Therapy in Oncology (Oncobell), IDIBELL, Catalan Institute of Oncology, L’Hospitalet de Llobregat 08908, Spain; Department of Information Technologies, Institut d’Investigació Biomèdica de Bellvitge (IDIBELL), L’Hospitalet de Llobregat 08908, Spain; Hereditary Cancer Program, Program in Molecular Mechanisms and Experimental Therapy in Oncology (Oncobell), IDIBELL, Catalan Institute of Oncology, L’Hospitalet de Llobregat 08908, Spain; Hereditary Cancer Program, Program in Molecular Mechanisms and Experimental Therapy in Oncology (Oncobell), IDIBELL, Catalan Institute of Oncology, L’Hospitalet de Llobregat 08908, Spain; Centro de Investigación Biomédica en Red de Cáncer (CIBERONC), Madrid, Spain; Department of Bioinformatics, Institut d’Investigació Biomèdica de Bellvitge (IDIBELL), L’Hospitalet de Llobregat 08908, Spain; Hereditary Cancer Program, Program in Molecular Mechanisms and Experimental Therapy in Oncology (Oncobell), IDIBELL, Catalan Institute of Oncology, L’Hospitalet de Llobregat 08908, Spain; Centro de Investigación Biomédica en Red de Cáncer (CIBERONC), Madrid, Spain

## Abstract

**Motivation:**

Germline variant classification allows accurate genetic diagnosis and risk assessment. However, it is a tedious iterative process integrating information from several sources and types of evidence. It should follow gene-specific (if available) or general updated international guidelines. Thus, it is the main burden of the incorporation of next-generation sequencing into the clinical setting.

**Results:**

We created the vaRiants in HC (vaRHC) R package to assist the process of variant classification in hereditary cancer by: (i) collecting information from diverse databases; (ii) assigning or denying different types of evidence according to updated American College of Molecular Genetics and Genomics/Association of Molecular Pathologist gene-specific criteria for *ATM*, *CDH1*, *CHEK2*, *MLH1*, *MSH2*, *MSH6*, *PMS2*, *PTEN*, and *TP53* and general criteria for other genes; (iii) providing an automated classification of variants using a Bayesian metastructure and considering CanVIG-UK recommendations; and (iv) optionally printing the output to an .xlsx file. A validation using 659 classified variants demonstrated the robustness of vaRHC, presenting a better criteria assignment than Cancer SIGVAR, an available similar tool.

**Availability and implementation:**

The source code can be consulted in the GitHub repository (https://github.com/emunte/vaRHC) Additionally, it will be submitted to CRAN soon.

## 1 Introduction

Cancer is a main public health problem and a leading cause of death (Siegel et al. 2022). Around 5%–10% of cancers worldwide are attributable to hereditary cancer (HC) syndromes (Nagy et al. 2004). HC patients harbour pathogenic germline variant(s) in cancer predisposition genes making them prone to develop multiple primary cancers at younger ages. Early identification of these individuals allows us to personalize their risk assessment, adapt their clinical follow-up, provide some targeted therapies, and offer cascade testing to relatives.

The use of next-generation sequencing (NGS) in diagnostics expands the number of genes analysed in a single test, increasing the diagnostic yield (Tung et al. 2016) but also the identification of variants of unknown significance (Lumish et al. 2017; Feliubadaló et al. 2019). Accurate variant classification is a huge challenge and a main burden of the incorporation of NGS into the clinical setting; only a correct classification allows proper genetic diagnosis and personalized risk assessment. Nowadays, variant classification is a time-consuming process that combines different type of evidence such as variant consequence, population frequencies, functional assays, *in silico* predictors, and co-segregation studies. Moreover, it is iterative due to continuous information updates and guideline refinement, enforcing periodic revisions of variant classification.

In 2015, the American College of Molecular Genetics and Genomics (ACMG) with the Association of Molecular Pathologists (AMP) published generic guidelines to standardize and provide an objective framework for evaluating variant pathogenicity in Mendelian disorders (Richards et al. 2015). However, some criteria proposed were qualitative and indefinite, allowing discrepancies in variant interpretation between laboratories (Amendola et al. 2016). Later, specific guidelines were published for some genes by collaborative groups or expert panels like Clinical Genome Resource (ClinGen) to adjust variant classification to gene and disease particularities (https://clinicalgenome.org/; accessed November 2022). Moreover, it was demonstrated that the criteria combination in ACMG/AMP guidelines was compatible with a quantitative Bayesian formulation (Tavtigian et al. 2018), which was later abstracted to a naturally scaled point system (Tavtigian et al. 2020). Additionally, CanVIG-UK consensus recommendations proposed some limitations to overlapping criterion combinations to avoid double counting of evidence (Garrett et al. 2021, https://www.cangene-canvaruk.org/_files/ugd/ed948a_f64f11f58e644521bc88f0b4ef1f5d01.pdf; accessed November 2022).

Different programmes have been developed to automatize variant classification by integrating different types of information. Most tools are based on ACMG/AMP general rules, like InterVar (Li and Wang 2017), PathoMAN (Joseph et al. 2017; Ravichandran et al. 2019), ClinGen Pathogenicity Calculator (Patel et al. 2017), CharGer (Scott et al. 2019), Varsome (Kopanos et al. 2019), or Franklin (https://franklin.genoox.com). Other tools focus on a set of genes like CardioClassifier (Whiffin et al. 2018) and CardioVai (Nicora et al. 2018) for inherited cardiac conditions or Cancer Predisposition Sequencing Reporter (CPSR) (Nakken et al. 2021) and Cancer-SIGVAR (Li et al. 2021) for cancer predisposition genes. CPSR uses SherLoc algorithm (Nykamp et al. 2017) that provided several refinements to the original guidelines. Cancer-SIGVAR uses a ClinGen update of ACMG/AMP guidelines (Abou Tayoun et al. 2018), considers ClinGen’s specific guidelines for *PTEN* (Mester et al. 2018), *CDH1* (Lee et al. 2018), RASopathies (Gelb et al. 2018), *RUNX1* (Luo et al. 2019) and hearing loss (Oza et al. 2018), and other sources (https://www.acgs.uk.com/media/11285/uk-practice-guidelines-for-variant-classification-2019-v1-0-3.pdf). However, specific guidelines or recommendations have been published for other HC genes like *ATM* (https://www.clinicalgenome.org/site/assets/files/7451/clingen_hbop_acmg_specifications_atm_v1_1.pdf; accessed November 2022), *CHEK2* (Vargas-Parra et al. 2020), *TP53* (Fortuno et al. 2021), and mismatch repair genes (MMRs) (https://www.insight-group.org/content/uploads/2021/11/DRAFT_Nov_2021_TEMPLATE_SVI.ACMG_Specifications_InSiGHT_MMR_V1.pdf; accessed November 2022). A deeper comparative of the above-mentioned tools can be found in Supplementary Table S1. Although automated tools aid the variant interpretation journey, the curator is still needed for proper integration of some clinical, genetic, functional, and literature information. Therefore, not all criteria can be fully automated.

Here, we introduce vaRiants in HC (vaRHC), an R package developed to automate, as far as possible, the variant classification process for HC genes. The Catalan Institute of Oncology is a monographic cancer centre, and our diagnostics laboratory is dedicated to HC testing. Accordingly, we aimed to increase accuracy in variant classification by automated collection and combination of data, and assignation of several criteria according to gene-specific guidelines for *ATM*, *CDH1*, *CHEK2*, *MLH1*, *MSH2*, *MSH6*, *PMS2*, *PTEN* and *TP53*, and the updated general ACMG/AMP rules for other cancer susceptibility genes.

## 2 Materials and methods

### 2.1 vaRHC package

The vaRHC package was conceived for the statistical computing environment R (v4.1.2), using functions from both R/Bioconductor and CRAN packages (more details in https://github.com/emunte/vaRHC).

### 2.2 Criteria analysis

ACMG/AMP original guidelines proposed 28 different criteria to evaluate pathogenicity or benignity. Not all criteria have the same strength: pathogenic criteria can be very strong (PVS1), strong (PS1–PS4), moderate (PM1–PM6), or supporting (PP1–PP5), whereas benign criteria can be standalone (BA1), strong (BS1–BS4), or supporting (BP1–BP7) (Richards et al. 2015). ClinGen later further specified these criteria. For instance, a decision-tree algorithm was developed to adapt PVS1 strength according to type and position of loss of function (LoF) variants (Abou Tayoun et al. 2018). PP5 and BP6 criteria, relying on reputable source classification without access to primary data, could lead to errors and double counting and were removed (Biesecker and Harrison 2018). Hence, many of the 28 criteria were changed, deleted, or extended to different weights depending on the gene.

Individual analysis of the criteria was performed to determine which could be fully automated, partially automated (requiring manual curation), not automatable, and which do not apply to some or all HC genes. General and gene-specific criteria used by vaRHC and their combinations are detailed in Supplementary Tables S2 and S3, respectively.

### 2.3 Criteria combination for variant classification

Tavtigian et al.’s (2020) naturally scaled point system was used to calculate the final classification of variants. In this approach, each pathogenic criterion achieving supporting, moderate, strong, or very strong strength sums 1, 2, 4, or 8 points, respectively, to the final score, and each criterion in favour of benignity subtracts these same values. The final score determines the classification of the variant (≥10: pathogenic; 6–9: likely pathogenic; 5–0: unknown significance; (−1)–(−5): likely benign; and ≤−6: benign).

### 2.4 Information retrieval and database

vaRHC leverages several existing databases and programmes. Due to their different conception and implementation, the information is retrieved from those sources in real time or queried in a local relational database implemented with MySQL (8.0.28-0ubuntu0.20.04.3 for Linux on x86_64).

#### 2.4.1 Local queries

Some source databases display stable information, with a renewal frequency of over 1 year. Hence, they are not queried real-time to decrease execution time and avoid possible issues due to their webpage maintenance. Instead, vaRHC is fed with a relational database (MySQL) gathering the information. The database is maintained at the Institut de Recerca Biomèdica de Bellvitge. Since SpliceAI and Provean predictors have recently retired their web-based or API version, the only way to obtain their scores is by downloading the software. To mitigate the difficulties that may cause installing extra software, several scores have been precalculated. The types of variants with precalculated scores and the complete information source list are found in Supplementary Methods.

#### 2.4.2 Real-time queries

Other databases, like ClinVar (Landrum et al. 2014, 2018) and InSiGHT (https://www.insight-group.org/variants/databases/; accessed November 2022) are updated weekly or monthly. To access the latest information, vaRHC queries them real-time via web scrapping, which it also uses to interrogate web interfaces from databases without download options. Online programmes without pre-computed databases are queried via REST API. The complete list of databases queried real-time is in Supplementary Methods.

#### 2.4.3 Customizable parameters

For easy customization, our local database contains a table with general and gene-specific cut-offs used for several criteria (BA1, BS1, PM2, PP3, BP4, and BP7). The user can provide vaRHC a txt file with custom values to change the default ones. See https://github.com/emunte/vaRHC/blob/main/data/gene_specific.txt to download a template.

### 2.5 Performance assessment

Classified variants from *ATM* (*n* = 32), *CDH1* (*n* = 279), *PTEN* (*n* = 139), and *TP53* (*n* = 118) were downloaded from the ClinGen evidence repository (https://erepo.genome.network/evrepo/, March 2022). For *CHEK2*, 13 variants classified in Vargas-Parra (2020) were used. An in-house dataset of 78 variants classified according to ‘ClinGen InSiGHT Hereditary Colorectal Cancer/Polyposis Variant Curation Expert Panel Specifications to the ACMG/AMP Variant Interpretation Guidelines Version DRAFT 1’ was used for MMR genes.

The comparison between vaRHC results and manual classification of the aforementioned variants was performed criterion by criterion, instead of only considering the final variant classification. Outcomes were grouped into nine scenarios for each criterion taken (Table 1).

**Table 1.** Labels assigned to the nine possible scenarios to quantitatively evaluate the performance of the programme.

Label	Description/scenario
Positive agreement	The criterion is assigned by both manual classification and vaRHC.
Negative agreement	The criterion is denied by both.
Previous version	The criterion used in the manual classification did not follow the most up to date guidelines for the gene.
Manual error	The manual choice clearly differs from the criterion statement in the guidelines.
Refined criterion	There is a discrepancy between the manual judgement and our software but in our view vaRHC’s output is more accurate.
Partially automated	There is a discrepancy due to the inability to fully automate the criteria.
Not assessed	When BA1 is assigned by manual classification, other criteria are sometimes not evaluated by manual classification, thus the performance of the additional criteria for that variant cannot be compared.
Not automated	The criterion has not been automated.
Not applicable	According to the guidelines, the criterion should not be applied.

### 2.6 Benchmark dataset

*CDH1* and *PTEN* variant datasets were also analysed with Cancer-SIGVAR (Li et al. 2021) using default settings. The number of differences between both software was statistically evaluated using the Kappa test from vcd (v 1.4-10) CRAN package; *P*-value was adjusted using Benjamini–Hochberg correction for multiple comparisons (Benjamini and Hochberg 1995). Significance for the adjusted *P*-value was 0.05.

## 3 Results

### 3.1 vaRHC package

The vaRHC package classifies single-nucleotide substitutions, deletions, and insertions up to 25-bp, intronic variants, and untranslated region variants.

**Figure 1:**
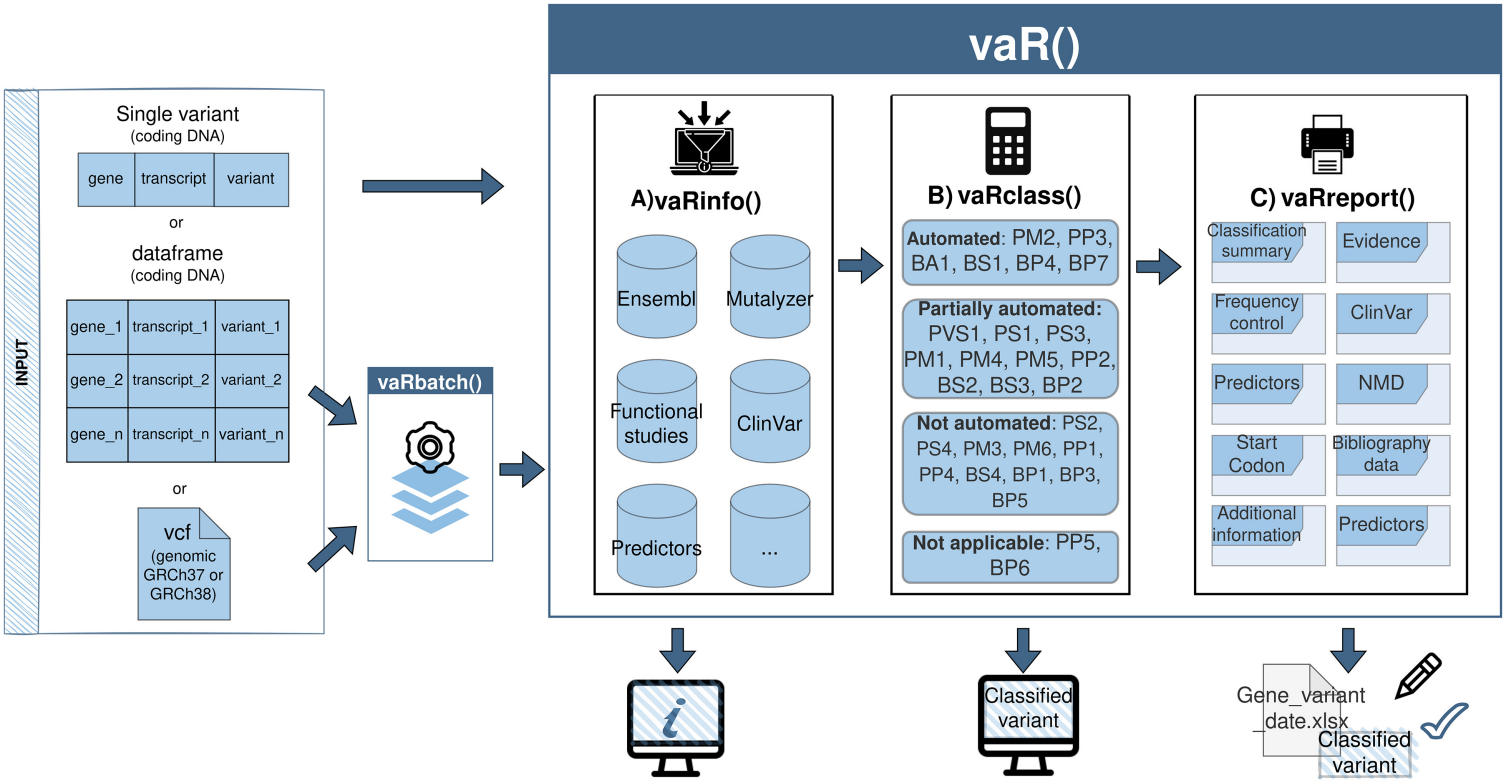
varRHC package: main functions and workflow. vaR() contains three functions as follows: (**A**) vaRinfo: retrieves variant information from distinct databases; (**B**) vaRclass: combines information to assign or deny ACMG criteria returning a final classification of the variant; and (**C**) vaRreport: prints the results in a spreadsheet (.xlsx) file. vaRbatch() allows to do the process sequentially.

Our package has a main function *vaR()* acting as a wrapper for three functions: *vaRinfo*, *vaRclass*, and vaRreport (Fig. 1). From the input of a gene, a transcript (RefSeq ID), and a variant name (in coding DNA nomenclature; http://varnomen.hgvs.org/), vaRinfo gathers relevant information from diverse sources. The second function vaRclass() uses the output of vaRinfo() to apply updated ACMG/AMP criteria considering gene specificities to calculate the different criteria met by the variant and explains the assignment or rejection of each criterion. Furthermore, it returns a final variant classification using Tavtigian’s Bayesian metastructure and most CanVIG-UK recommendations (Supplementary Table S3). Lastly, vaRreport() generates a user-friendly .xlsx file to examine and store results, allowing non-bioinformatic users to work with them and modify the file, adding their considerations or information regarding non-automatable criteria.

For users wanting to classify variants in batch, vaRbatch() function has been created to interrogate vaR() function sequentially. The input can be either a dataframe with variants in coding DNA nomenclature or a variant call format file. The latter can be based on the GRCh37 or GRCh38 genome assemblies and will be annotated in coding DNA, considering MANE select transcript or Locus Reference Genomic (LRG) t1 transcripts depending on user specifications. Moreover, each time it is executed a new log file is created. This provides an accurate per variant time-execution registry and collects all possible errors encountered during the process.

The current package works properly for the main HC genes (*n* = 53). It was also tested for all genes with a LRG entry (*n* = 1325) and it works for most of them (listed in Supplementary Table S4). It should also work for most genes with a MANE select transcript, although not all of them have been tested. However, variants located at positions where the reference allele differs between GRCh37 and GRCh38 cannot be computed by vaRHC. Nevertheless, these variants are usually polymorphisms that can be classified as benign based only on their high frequency in population datasets. The package relies on Mutalyzer v3 for variant nomenclature correction. Since not all transcript versions are supported by Mutalyzer, the tool searches for an available version and returns a warning to inform the user.

### 3.2 Performance assessment

To evaluate vaRHC’s performance, 659 variants previously manually classified using specific guidelines were selected, of which 20 (3%) were not supported by vaRHC due to their nature (complex deletion–insertions, deletions/duplications >25 bp, inversions); thus, 639 variants were finally assessed: 29 *ATM*, 274 *CDH1*, 13 *CHEK2*, 13 *MLH1*, 22 *MSH2*, 33 *MSH6*, 10 *PMS2*, 128 *PTEN*, and 117 *TP53*.

#### 3.2.1 Criteria assignation performance

**Figure 2:**
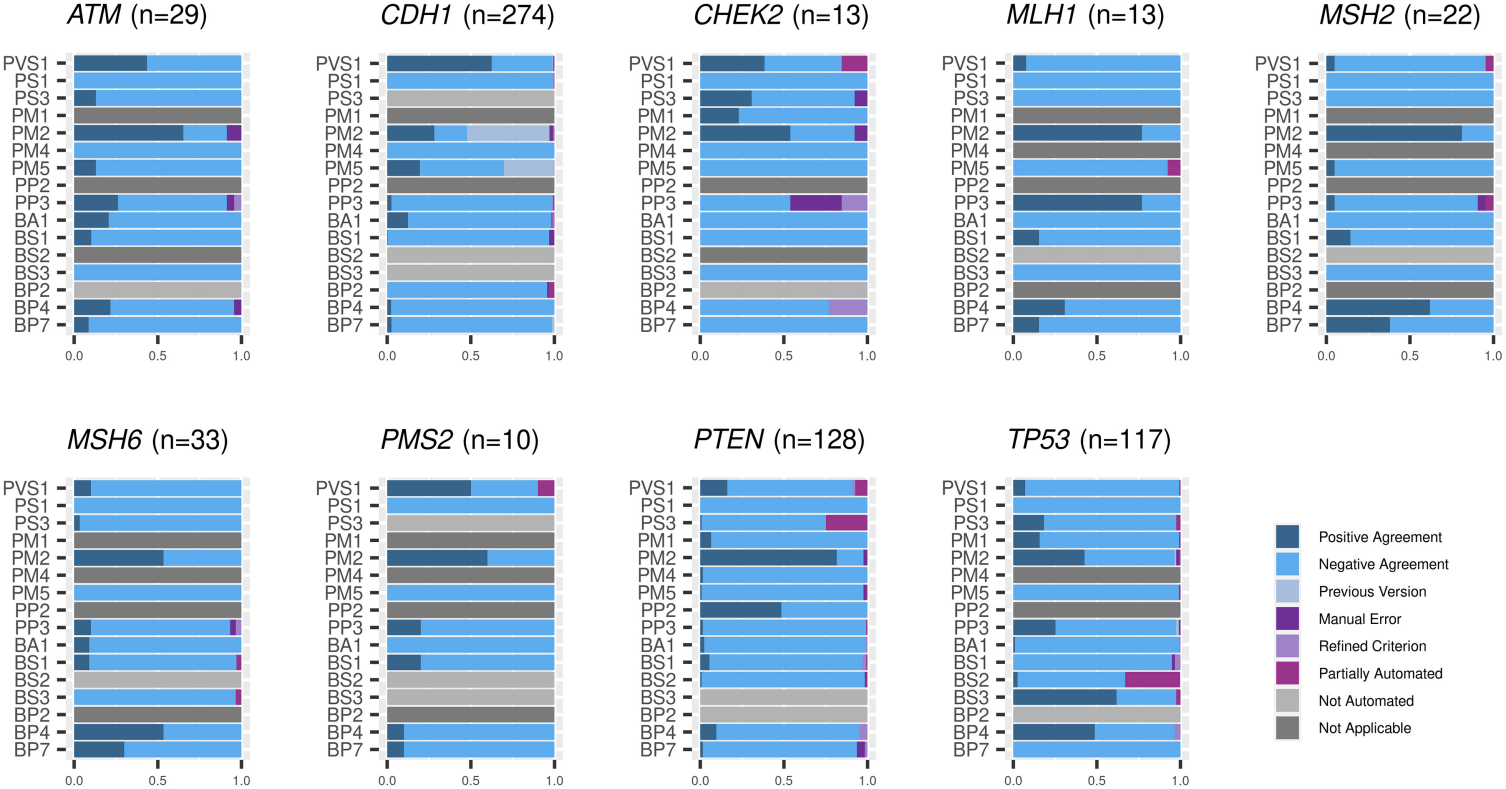
Tool performance assessed by gene and criteria. Stacked bar diagrams show the proportion of variants falling within each scenario (Table 1), labelled with a different colour (see legend in the figure). Each gene and criterion is evaluated separately (only fully or partially automated ones). Maximum number of variants analysed per gene is in parenthesis and corresponds to variants evaluated for BA1 and BS1. Since BA1 is a standalone criterion (incompatible with BS1), when BA1 was assigned by manual classification, the remaining criteria were not assessed in most variants and omitted from the comparison.

The tool’s performance was compared with the previous classification, showing that each fully automated criterion is correctly assigned in at least 97.7% of the variants. Figure 2 shows the performance broken down by gene and criteria (details in Supplementary Table S6). Supplementary Tables S7–S12 include all variants used for validation and a detailed explanation of discordant criteria (labelled according to Table 1).

Below, the validation results are depicted according to the different criteria groups.

##### 3.2.1.1 Population data (criteria codes BA1, BS1, BS2, PS4, and PM2)

The gnomAD v2.1.1 dataset was chosen to assess the variant allele frequency in control populations (Karczewski et al. 2020). Although v2 gathers fewer genome sequences than v3, it contains many more exome sequences (the main regions of interest for diagnostics), reaching a much higher allele number (±250 000) and increasing its statistical power. These databases were not aggregated as they overlap and it would distort allele frequencies (Gudmundsson et al. 2022). Specifically, the non-cancer dataset (minimum coverage of 20×) was selected (details in Supplementary Methods).

Furthermore, following ENIGMA recommendations (https://enigmaconsortium.org/library/general-documents/enigma-classification-criteria/; accessed November 2022), for BA1 and BS1, gnomAD founder populations, like Ashkenazi Jewish and Finnish, were omitted as bottleneck effects could mask natural negative selection of pathogenic variants. This exception was not considered in ClinGen; thus, disagreements were labelled as ‘refined criteria’.

Positive and negative agreements were found in 98.9% of variants for BA1 and in 96.9% for BS1. The refined criterion accounted for 0.63% of variants for BA1 and 1.10% for BS1. When manual ClinGen classifications used gnomAD v3, Exome Aggregation Consortium, or 1000 Genomes as population datasets to assign BA1 or BS1, these were conservatively labelled as ‘partially automated’ and accounted for 0.16% and 0.47% of variants, respectively.

Some gene-specific guidelines require the use of the lower confidence interval (CI) limit of allele frequencies from population datasets to compare them with maximum credible allele frequencies for a pathogenic variant to assign BA1 or BS1. Nevertheless, the corresponding manual classification dataset has not always applied it (see Supplementary Table S8). These manual errors explain the remaining 0.31% and 1.57% for BA1 and BS1, respectively.

Regarding the PM2 criterion, positive and negative agreements were found in 77.2% of variants. The main reason for disagreements was that some ClinGen pilot variants were classified using previous versions of gene-specific guidelines, lacking recent specifications, as vaRHC does. For example, for *CDH1*, PM2 was downgraded in version 3 to supporting strength, but many variants in the repository remain as moderate. These variants account for 20.0% of the total and were labelled as ‘previous version’.

Only two variants (0.34%) were not assigned because they were only present in the gnomAD v3 non-cancer dataset (partially automated). Manual errors account for 2.2% of cases, many consisting of input variant nomenclature mistakes (e.g. using information from another variant or not finding the variant in the gnomAD database, although it was there). For HC susceptibility genes, gnomAD non-cancer is a more accurate population dataset, thus 0.2% of the variants were labelled as refined criterion. The BS2 criterion does not apply to *ATM* and *CHEK2* guidelines (6.2%) and is not automated for *CDH1* and MMR genes (52.9%). The lack of databases sharing this information impedes full automation of this criterion. Consequently, positive agreements only accounted for 0.69% of cases and were based on information from the FLOSSIES database and, for some genes, homozygote status from the gnomAD v2.1.1 non-cancer dataset. Negative agreements represented a 33.4%. ClinGen expert panels assign BS2 to 6.7% of variants thanks to in-house datasets (not shared), the literature, or ClinVar comments, which are not easily automatable. Only one variant (0.17%) was labelled as manual error (see Supplementary Table S11).

The PS4 criterion is not applicable to MMR genes or relies on information not stored in public databases, thus is not automated for other genes.

##### 3.2.1.2 Computational and predictive criteria (PVS1, PS1, PM4, PM5, PP3, BP4, and BP7)

**Figure 3:**
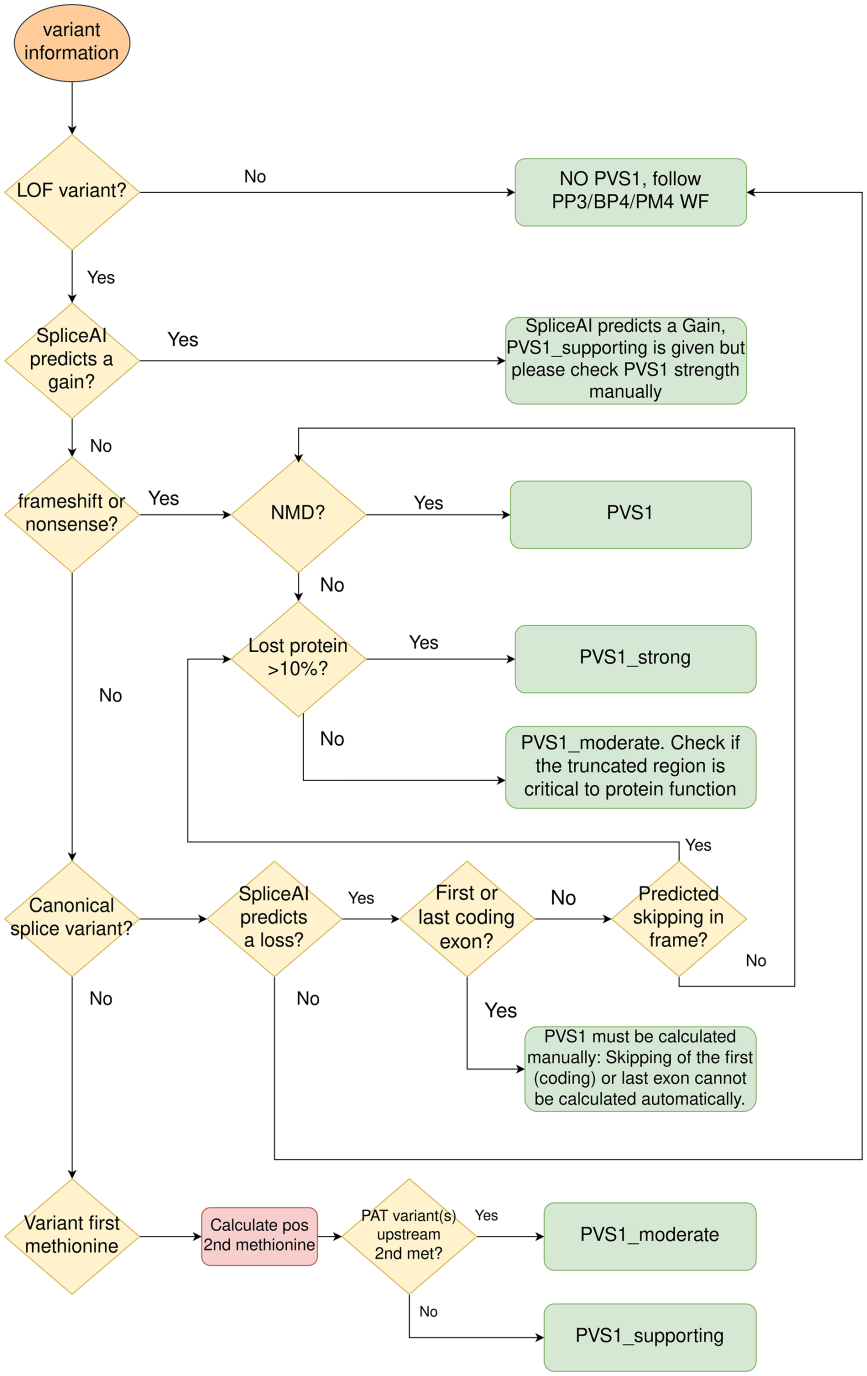
Flowchart showing the algorithm implemented in the vaRHC package to assign different PVS1 criterion strengths to LoF variants, based on ClinGen recommendations (Abou Tayoun et al. 2018).

For PVS1, the programme follows an algorithm based on Tayoun’s decision tree (Abou Tayoun et al. 2018) for general classification and incorporates gene specificities where there are specific guidelines (Fig. 3). As shown in the figure, it integrates the splicing prediction, as a splicing alteration would affect the variant consequence.

Positive and negative agreements represented 96.7% of variants for PVS1 and 2.7% were labelled as ‘partially automated’. The limitation in automation was mostly due to difficulty in determining splicing outcome when spliceAI predicts a splice site gain. Thus, vaRHC is conservative, assigning a supporting strength and returning a warning message suggesting an RNA test before assigning a higher strength. Likewise, when exon skipping is predicted for canonical splice variants located at the first or last exon, vaRHC returns a warning with no PVS1 strength assigned.

*PTEN* guidelines do not incorporate Tayoun’s algorithm, only assigning a very strong strength. As a refined criterion, vaRHC considers modified Tayoun workflow for this gene (but incorporates the specific guideline consideration that truncating variants 5′ to c.1121 must be assigned as very strong); this changes the PVS1 strength assigned to two *PTEN* variants (0.34%).

Furthermore, variant c.1137 + 1delG in *CDH1* was assigned as very strong by ClinGen. However, per site-specific recommendations in the splicing table in version 3.1, it should be downgraded to strong. Thus, the variant was classified as ‘previous version’ (0.17%).

Regarding prediction criteria (PP3 and BP4), only some guidelines specify which to use and their cut-offs. For the other genes, as general guidelines, REVEL metapredictor was adopted to predict protein impact, with optimized cut-offs proposed by Cubuk (2021). SpliceAI (Jaganathan et al. 2019) was selected as the main splice predictor. Since no cut-off was previously established, 518 RNA-tested variants were analysed to set PP3 and BP4 thresholds (Supplementary Methods and Supplementary Table S13). The final cut-offs for spliceAI were ≥0.5 for PP3 and ≤0.15 for BP4, giving pathogenicity odds ratio values of 25.3 and 0.037 for PP3 and BP4, respectively. Per Tavtigian’s Bayesian framework, these could account for PP3_Strong and BP4_Strong, but a supporting strength was conservatively maintained in the tool.

Only MMR gene splice defect prediction combines SpliceAI with other algorithms from http://priors.hci.utah.edu/PRIORS, as specified in their guidelines. The retrieved predictors and cut-offs for each gene are in Supplementary Table S14.

From the above data, positive and negative agreement represented 96.7% of variants for PP3 and 97.5% in for BP4. For PP3, two *TP53* variants were affected by changes in predictor cut-offs between specific guideline versions and thus classified as ‘previous version’ (0.34%). Manual errors in PP3 were mostly due to applying PP3 when PVS1 or PM1 (by functional domain) was also assigned. According to CanVIG-UK, these criteria should not be combined as the same information is used to calculate them. Another manual error arises from mistakes in variant information queries. Manual errors represented 1.37% in PP3 and 0.18% in BP4. Discrepancies due to lack of detail in specific guidelines, which led us to choose REVEL and SpliceAI, were considered ‘refined criterion’ and accounted for 0.86% in PP3 and 2.29% in BP4. Four variants (0.69%) were categorized as partially automated for PP3 (see Supplementary Tables S8, S10, and S11).

The BP7 criterion was classified as positive or negative agreement in 98.1% of variants. In *CDH1*, three variants were labelled as ‘previous version’, since manual classification did not assign BP7 to them, for being in a highly conserved nucleotide, a condition no longer applied. Manual errors correspond to six variants in *PTEN* (1.03%) where the nucleotide is predicted to be strongly conserved by Phastcons (value = 1) but manual classification assigned BP7 anyway. Moreover, Phylop was also assessed to determine nucleotide conservation (when required). Some scores obtained with the tool did not match those from manual classification. Three variants in *CDH1* were manually classified according to previous versions (0.51%) and two *PTEN* variants as refined criteria (0.34%) (see Supplementary Table S11).

Concerning PS1 and PM5, variants in the same codon as the test variant must be also classified according to gene-specific guidelines. Moreover, some guidelines, such as *TP53*, require that the variant be specifically classified by the ClinGen expert panel. To ensure this, vaRHC uses information from ClinVar variants classified by an expert panel (i.e. ClinGen for HC genes). Furthermore, these criteria were modified over time in *CDH1* guidelines: PS1 no longer applies and PM5 uses a new criterion applying to non-sense and frameshift variants predicted or proven to undergo NMD and to some canonical splicing variants.

It was difficult to validate vaRHC for PS1 and PM5 since the criteria dictates to compare test variants with expert panel classified variants. This is ClinGen dataset, the same that we are using to do the performance assessment. As expected, there were few pairs of variants at the same codon, both classified by the expert panel. Therefore, no variant was assigned PS1 by the software, and 116 of 121 variants assigned PM5 were due to the new *CDH1* criterion for truncating variants rather than the classic variant comparison criterion. To reliably validate the tool’s performance, a list of 37 hypothetical variants located at the same codons as the variants used in the primary validation was created (see Supplementary Methods and Supplementary Table S15). The programme assigned PS1 or PM5 to 19 variants; PS1 and PM5 were denied in the remaining 18 variants as some criteria requirements were not met.

##### 3.2.1.3 Functional data (PS3, PM1, PP2, BS3, and BP3)

Functional data were generally refractory to automatic extraction. However, publication of some mid-to-high throughput clinically calibrated functional assays allowed their incorporation as an innovative feature of vaRHC. Most articles listed in gene-specific guidelines and some accomplishing the experimental conditions demanded in Brnich et al. (2019) to assign PS3 or BS3 were collected in the database (see Supplementary Methods, Section 1.1). Consequently, 5.1% of variants were assigned PS3 and 12.2% BS3. No literature information was used to assign PS3/BS3 to *CDH1* and *PMS2* genes (42.0%), or BS3 to *PTEN* (63.2%). Criteria were assigned only by manual classification in 5.8% of variants for PS3 and 0.69% for BS3.

Additionally, a search string combining different names for the variant was provided to be used in Internet search engines; this can help users find the most relevant articles for functional, allelic, and clinical criteria.

The PM1 criterion does not apply *to ATM*, *CDH1*, and MMR genes (56.8%). Only one variant (c.892G>T in *TP53*, 0.17%) was labelled as ‘manual error’ since manual classification assigned it PVS1 and PM1. Per CanVIG-UK, they should not be combined because PM1 can only be used for missense variants and small in-frame deletions and insertions. The remaining 43.0% correspond to positive and negative agreements.

Per specific guidelines, the PP2 criterion should only be used for *PTEN*, being correctly assigned in all cases.

##### 3.2.1.4 Allelic data (PM3 and BP2)

PM3 was not automated as allelic data in patients are seldom collected in databases. The BP2 criterion does not apply to MMR genes (12.7%) and can only be partially automated for *CDH1*. Specifically, only supporting strength can be assigned when the variant is in homozygosity in gnomAD since no public database of individuals without personal and/or family history of associated tumours was found. Manual classification assigned BP2_Strong to some variants as they were homozygous in a control cohort (gnomAD v2.1.1). However, according to Harrison et al. (2019), individuals in gnomAD should be cautiously considered as general population instead of healthy individuals for adult-onset conditions. Thus, 0.3% of variants were categorized as ‘manual errors’.

##### 3.2.1.5 Other databases (PP5 and BP6)

According to ClinGen, reputable source not linked to the supporting evidence should not be used as criteria thus PP5 and BP6 should not be applied (Biesecker and Harrison 2018).

##### 3.2.1.6 Segregation data (PP1 and BS4), de novo data (PS2 and PM6), and other data (PP4 and BP5)

Due to the current lack of segregation information in databases, none of these criteria could be automated.

#### 3.2.2 Automated classification performance

vaRHC classified each variant in 15–120 s. Automated classification concorded with manual classification in 63.4% of variants with five-tier classification, increasing to 74.0% with three-tier classification (Supplementary Table S5). Users would be expected to add non-automatable criteria to reach a final classification.

### 3.3 Benchmark

**Figure 4:**
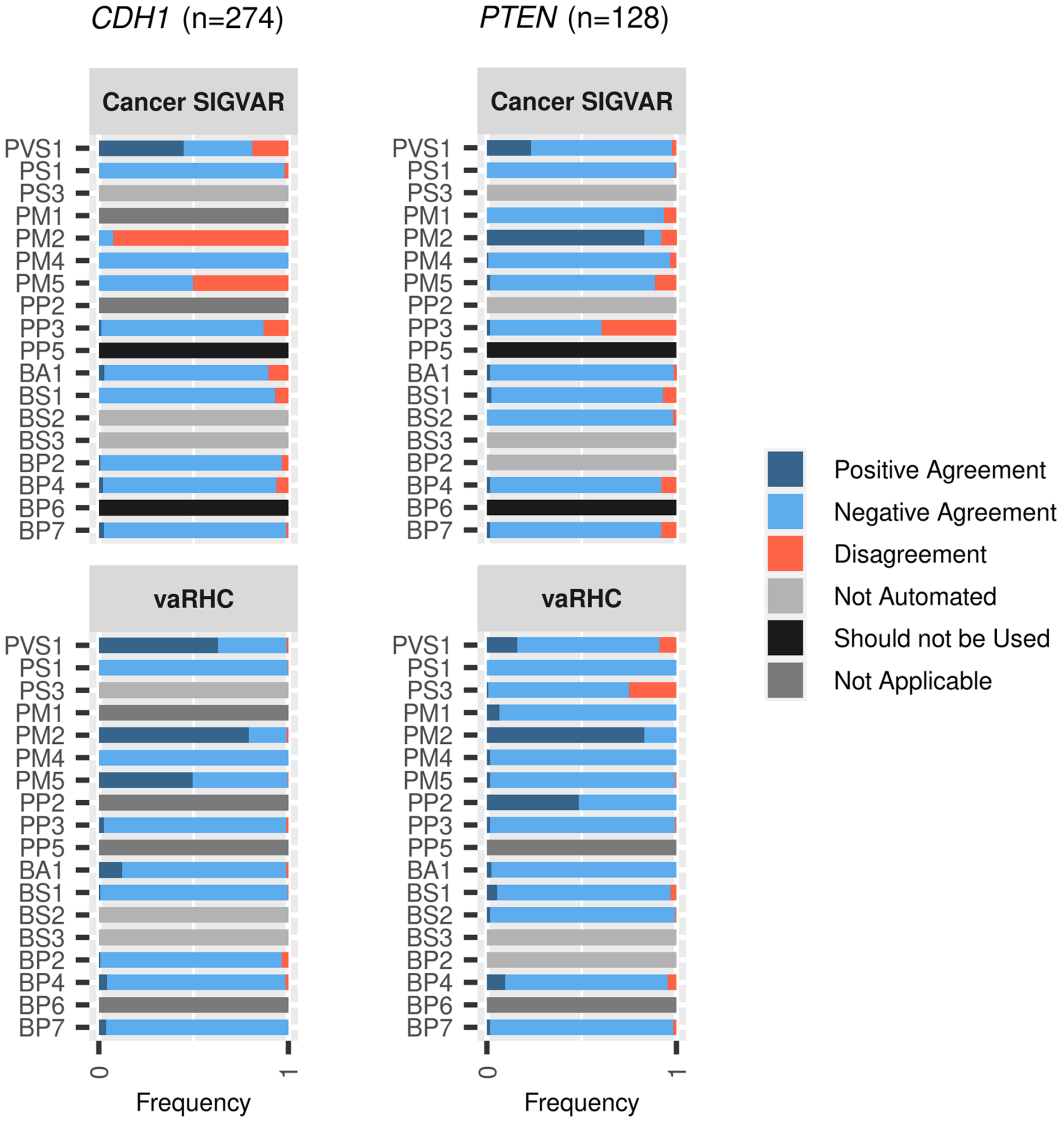
Performance of Cancer-SIGVAR and vaRHC in comparison with the modified benchmark dataset. Stacked bar diagrams show the proportion of variants falling within each scenario (see Sections 3, 3.3, and Table 1), labelled with a different colour (see legend in the figure), for CDH1 and PTEN genes. The maximum number of variants analysed per gene is in parenthesis. This corresponds to variants evaluated for BA1 and BS1. Since BA1 is a standalone criterion (incompatible with BS1), when BA1 was assigned by manual classification, the remaining criteria were not assessed in most variants and omitted from the comparison.

Cancer-SIGVAR (Li et al. 2021) is a free web tool (http://cancersigvar.bgi.com) based on ACMG/AMP rules and focused on interpreting HC variants. We analysed, criteria by criteria, the performance of Cancer-SIGVAR and vaRHC for *CDH1* and *PTEN* variants against the ClinGen repository (the same as in the validation dataset). However, in the vaRHC previous performance assessment we had identified manual errors and a proportion of variants classified without updated guidelines (see Fig. 2 and Supplementary Tables S7–S12). To address this, some ClinGen criteria assignments were modified, correcting variants labelled as ‘manual errors’ and ‘previous version’. Conservatively, variants categorized as ‘refined criterion’ were not altered. The performance of both tools against this modified benchmark dataset was compared in (Fig. 4).

As it can be seen in the figure, vaRHC does not use PP5 and BP6 criteria, as dictated by ClinGen (Biesecker and Harrison 2018), it incorporates the updated v3 CDH1 guidelines (September 2021) and also data from functional assays. Due to these and other assets, vaRHC improves the performance of Cancer-SIGVAR.

Cohen’s Kappa test comparing cancer-SIGVAR and vaRHC revealed significant differences for PM2 (kappa = 0.06, *P*-value = 2.98*E*−06), PM5 (kappa = 0.33, *P*-value = 1.34*E*−101), PP3 (kappa = 0.32, *P*-value = 9.91*E*−05), and BP4 (kappa = 0.47, *P*-value = 1.72*E*−07) criteria in *CDH1* and PM1 (kappa = 0.48, *P*-value = 0), PM4 (kappa = 0.42, *P*-value = 3.08*E*−02), PM5 (kappa = 0.30, *P*-value = 2.63*E*−02), BP4 (kappa = 0.42, *P*-value = 4.49*E*−10), and BP7 (kappa = 0.35, *P*-value = 6.58*E*−03) in *PTEN*. In contrast, comparing vaRHC with the modified benchmark dataset, all these criteria obtained Kappa values >0.7 (see Supplementary Table S16).

## 4 Discussion

Variant classification is a main challenge and bottleneck in the genetic testing process using NGS. ClinGen and expert panels work on adapting generic ACMG/AMP guidelines has led to several gene-specific recommendations, adding difficulty to the already long and complex manual classification process. Thus, the development of automated tools could assist this process, accelerating it and reducing manual error. However, most tools available use original ACMG/AMP general recommendations and few are adjusted to gene-specific ones.

Here, we present vaRHC, an R package developed to cover these needs. It automates as much as possible the variant classification process: it collects pieces of information from several databases and it combines them to assign or deny criteria according to the most up-to-date guidelines. To support classification in a more meaningful clinical class, vaRHC leverages Tavtigian’s Bayesian approach and it also integrates most CanVIG-UK recommendations to finally assign or deny criteria, thus avoiding combining overlapping criteria, evading double counting. Moreover, vaRHC can be easily incorporated into bioinformatic pipelines, and adds a downloadable output as an editable, user-friendly spreadsheet (.xlsx) file. This function allows variant curators unfamiliar with the R environment to work with the data and add extra considerations, like the refinement of automated criteria or information derived from non-automatable criteria. Our validation demonstrates the robustness of the software developed in assigning automated criteria supporting the notion that automation tools are valuable in the variant classification process. However, they should not substitute the crucial role of the variant curator in supervising automated criteria, reviewing and incorporating data from the literature and in-house databases to assign semi-automatable and non-automatable criteria.

Despite ACMG/AMP and ClinGen’s efforts to standardize guideline criteria to provide an objective framework, some ambiguous criteria remain that could cause discrepancies between laboratories. When identified we have labelled them as ‘refined criteria’. Examples are the use of the non-cancer dataset and outbred subpopulations to calculate criteria related to population data or the choice of predictors and/or thresholds when the criteria do not establish them. Particularly, SpliceAI was selected as the unique splicing predictor, except for MMR genes, where Prior (UTAH) was also used according to gene-specific guidelines. Some guidelines suggest variant scoring using a consensus of two or three predictors. However, it was recently demonstrated that this provides little benefit (Wai et al. 2020). Moreover, a benchmark recommended the use of SpliceAI because it has the highest area under the curve (Riepe et al. 2021). To establish cut-offs for benignity and pathogenicity, a specific assessment was designed with 518 RNA-tested variants. Of note, vaRHC uses all proposed thresholds as defaults but is easily modifiable with a txt file.

Previous tools have almost exclusively focused on the final classification of the variant instead of analysing criterion by criterion. As automated tools lack some information derived from non-automatable criteria, many variants are classified as ‘unknown significance’. To palliate this, some programmes are less strict in assigning some criteria (e.g. permissive population frequency thresholds for BA1, BS1, and PM2 by software like Varsome) or use criteria that currently do not apply (e.g. PP5 and BP6 by SIGVAR, InterVar, Varsome, Franklin, Pathoman, and CharGer) to leverage ClinVar classification information and artificially approach its result. We recommend to use these tools in a research context to prioritize variants rather than directly employ them in diagnostics. One of vaRHC’s strengths is that it is strict in assigning criteria. Moreover, for each denied or assigned criteria, it always returns an explanation and, when appropriate, suggests extra considerations that should be noted for manual curation of criteria. This makes vaRHC suitable for use in molecular diagnostics units. In fact, the package is currently used by the Catalan Institute of Oncology (ICO) Molecular Diagnostics Service.

Although vaRHC was developed to answer to HC genetic testing needs, its approach to classify variants in HC genes without gene-specific guidelines is generalizable to most genes where loss-of-function variants cause heritable diseases. Furthermore, the customizable nature of most parameters allows users to adapt the tool to their needs. The use of the gnomAD v2.1. non-cancer dataset for variant population frequency assessment is compatible with other diseases, this dataset has only subtracted a few samples belonging to cancer patients, that could have invalidated conclusions for variants involved in these conditions and would not have increased substantially the power of the datasets.

The performance of our tool was compared with the ClinGen datasets for each HC gene with specific ClinGen guidelines. These datasets have a limited size compared with ClinVar, but they are manually curated by experts and inform the assignation of each criterion, which cannot be matched by the ClinVar dataset. Although a more homogeneous dataset would be desirable, each ClinGen Variant Curation Expert Panel chose the number of variants for its curated set as appropriate to exemplify the use of its criteria. We have expanded this collection with manually curated *CHEK2* variants from an article that ‘proposes’ *CHEK2*-specific rules and with MMR gene variants from the in-house ICO diagnostics laboratory DB. Our tool and Cancer-SIGVAR were compared with the ClinGen dataset only for *CDH1* and *PTEN*, since those are the genes where Cancer-SIGVAR follows gene-specific guidelines. Cancer-SIGVAR incorporates specific guidelines for some genes but does not cover recent ClinGen guidelines for relevant cancer genes. For impartiality, variants labelled as ‘manual errors’ and ‘previous version’ in ClinGen were corrected. Besides the aforementioned differences in using non-applicable criteria, the Kappa Test analysis revealed significant differences in other criteria assigned, favouring vaRHC.

A limitation of vaRHC is that, currently, it does not work for all variant types or lengths. Its execution time is around 15–30 s per variant, but can reach 2 min for insertions and deletions, where SpliceAI cannot be precomputed. Furthermore, its connection to ClinVar and other databases relies on the good performance and connectivity of their websites. Moreover, perpetual maintenance is planned and needed because any change in the html structure or content of queried websites via web scrapping can lead to different types of errors. Also, the database contains information on some published functional assays, but this is not due to a self-renewing ability to mine the literature, but to a manual effort. Nevertheless, the current vaRHC release plan includes the addition of recently published functional assays. Also, updates will include multiple features such as using gnomAD v3 information and downloading the report in other file extensions.

In summary, the performance assessment and benchmark carried out in the present work corroborates the robustness and excellent performance of vaRHC to assist variant classification. To our knowledge, our package outperforms tools available for several reasons: (i) it is the first freely available R package that semi-automates the process; (ii) it uses Tavtigian’s Bayesian metastructure nuanced by CanVIG-UK criterion combination rules, and (iii) it includes gene-specific guidelines for several commonly studied cancer genes, like *ATM*, *CDH1*, *CHEK2*, *MLH1*, *MSH2*, *MSH6*, *PMS2*, *PTEN*, and *TP53*. Altogether, we expect that vaRHC will facilitate the task of variant curators in clinical settings by reducing time for variant classification, limiting manual errors, and allowing the personalization of some parameters according to clinical and laboratory data.

## Supplementary Material

btad128_Supplementary_Data
