# Supplementary material for: vaRHC: an R package for semi-automation of variant classification in hereditary cancer genes according to ACMG/AMP and gene-specific ClinGen guidelines
Source: Bioinformatics. 2023 Mar 14;39(3):btad128. doi: 10.1093/bioinformatics/btad128 (PMC10032633; doi:10.1093/bioinformatics/btad128)
Supplement: btad128_Supplementary_Data [file btad128_supplementary_data.zip › btad128_Supplementary_Data/SupplementaryMethods13102023.docx]

**Supplementary Methods**

**1. Databases**

**1.1 Local queries**

The database created contains Locus Reference Genomic (LRG) stable reference sequences for clinical reporting (<https://www.lrg-sequence.org/>; accessed March 2022) from a set of cancer genes listed in Supplementary Table 3. It also stores their transcript and genomic information with Ensembl transcript ID, NCBI RefSeqs, and the corresponding consensus coding sequence (CCDS). The user can execute vaRHC for variants in other cancer and non-cancer genes and different transcripts, but those stored in the database have been tested for bugs.

The database also contains information on variant frequency in the large population datasets gnomAD (v2.1.1) non-cancer and non-neuro (Karczewski *et al.*, 2020), large scale multifactorial likelihood quantitative analysis of *BRCA1* and *BRCA2* variants (Parsons *et al.*, 2019), and functional studies for *ATM* (Scott *et al.*, 2002; Barone *et al.*, 2009; Mitui *et al.*, 2009), *BRCA1* (Lyra *et al.*, 2020), mismatch repair genes (MMR) (Drost *et al.*, 2019, 2020; Jia *et al.*, 2021; Rayner *et al.*, 2022), *TP53* (Kato *et al.*, 2003; Giacomelli *et al.*, 2018; Kotler, Segal and Oren, 2018), *PTEN* (Trojan *et al.*, 2001; Agrawal, Pilarski, and Eng, 2005; Chen *et al.*, 2017) and *CHEK2* (Wu, Webster and Chen, 2001; Wu *et al.*, 2006; Schwarz, Lovly, and Piwnica-Worms, 2003; Sodha *et al.*, 2006).

Moreover, it stores *in-silico* predictor databases such as the HCI Database of Prior Probabilities of Pathogenicity for Single Nucleotide Substitutions protein level information (<http://priors.hci.utah.edu/PRIORS/>; accessed November 2022) and dbnsfp, a database developed for functional prediction and annotation of all potential non-synonymous, single-nucleotide substitutions in the human genome. From dbnsdfp, varHC obtains predictions of REVEL, VEST4, PROVEAN, BayesDel (Liu *et al.*, 2020), Align_gvgd Zebrafish, and Provean no AF for *TP53.*

Finally, it also includes valuable site-specific information extracted from gene-specific guidelines. For instance, the splicing effects table for canonical variants in *CDH1,* sequence of the first six bases of the intron to assign or deny PVS1 to G>non-G for last base of exon variants for MMR and ATM.

**1.2 Real-time queries**

The following databases update information periodically: ClinVar aggregates information about human variations and their relationship to phenotypes (Landrum *et al.*, 2014, 2018) and InSiGHT collects information about DNA variants from genes related to gastrointestinal cancer (<https://www.insight-group.org/variants/databases/>)(*InSiGHT variants databases - InSiGHT*, 2022). These databases are queried via web scrapping so as to have their latest version.

vaRHC queries some software that do not have a pre-computed database for all variants, such as Mutalyzer (Lefter *et al.*, 2021) or Variant Effect Predictor (VEP) (McLaren *et al.*, 2016) and web interfaces that do not provide the option to download data, such as the Fabulous Ladies Over Seventy (FLOSSIES) database (<https://whi.color.com/>). Online tools without pre-computed databases are queried via REST API. For example, base-wise conservation in the vertebrate Multiz Alignment & conservation (100 species), Phastcons, and Uniprot values/data are obtained from UCSC API (<https://genome.ucsc.edu/goldenPath/help/api.html>).

Although SpliceAI provides a pre-computed database, it only considers the author’s default parameters (Jaganathan *et al.*, 2019). However, increasing the ”Max distance” window allows assessing the effect of the variant on the score of more distant positions. Calculating SpliceAI Δscores for a variant is time-consuming using REST API (<https://spliceailookup-api.broadinstitute.org/>). To reduce the number of queries and thus time spent, the masked Δscores using a window of 1000 nucleotides have been previously calculated and stored in our database for all possible exonic substitutions in *ATM, CHEK2, MLH1, MSH2, MSH6, PMS2, PTEN*, and *TP53.*

**2. GnomAD dataset pre-processing**

The GnomAD non-cancer dataset was selected as a general population control with a minimum coverage of 20x. Sequenced exomes and genomes in v2.1.1 belong to different individuals. Thanks to this, the total allele number and allele count is considered jointly (if both datasets reach the minimum coverage). When a variant is not found in exomes or genomes, GnomAD does not provide its allele number (total number of called high genotypes at its position). The omission of this information can lead to a false allele frequency calculation for the whole dataset. To mitigate this issue, vaRHC adopts the following approach: it uses the allele number of the nearest upstream and downstream variant in a window of up to 50 bp and adopts its allele number (if it finds both, it calculates the mean). When no variant is found, the program does not calculate criteria related to population frequencies.

**3. Splicing predictor assessment and cut-off selection**

***3.1. Variant selection***

A dataset of 518 RNA-tested variants with unequivocal splicing results was used to establish cut-offs for benignity (BP4) and pathogenicity (PP3). Variants were obtained from the literature (Menéndez *et al*., 2012; Thomassen *et al*., 2012; Colombo *et al*., 2014; Whiley *et al*., 2014; Quiles *et al*., 2016; Rofes *et al*., 2020) and belonged to genes (number of variants in parenthesis): APC-NM_001354896.1 (2), ATM-NM_000051.3 (9), BRCA1-NM_007294.3 (167), BRCA2-NM_000059.3 (161), BRIP1-NM_032043.2 (3), CDH1-NM_004360.3 (12), CHEK2-NM_007194.3 (3), DKC1-NM_001363.3 (1), FBN1-NM_000138.4 (9), FGFR1-NM_001174067.1 (1), FLNB-NM_001457.3 (1), KANSL1-NM_015443.3 (1), MED13L-NM_015335.4 (1), MEF2C-NM_002397.4 (1), MLH1-NM_0002492 (74), MSH2-NM_000251.2 (25), MSH6-NM_000179.2 (9), MUTYH-NM_001128425.1 (1), MYBPC3-NM_000256.3 (1), NGLY1-NM_018297.3 (1), PAFAH1B1-NM_000430.3 (1), PALB2-NM_024675.3 (8), PIGB-NM_004855.4 (1), PMS2-NM_000535.5 (2), PNKP-NM_007254.3 (1), POLD1-NM_001308632.1 (1), PTCH1-NM_000264.4 (1), PTEN-NM_000314.4 (6), RAD51D-NM_002878.3 (1), SF3B4-NM_005850.4 (1), SKI-NM_003036.3 (1), SMAD4 - NM_005359.5 (1), STK11-NM_000455.4 (2), TP53-NM_000546.5 (7), TSC-NM_000368.4 (1), and TSC2-NM_000548.3 (1).

Variants affecting splicing (n=317) were divided into four groups depending on their consequence: 21 caused an acceptor gain (AG), 25 a donor gain (DG), 94 an acceptor loss (AL), and 177 a donor loss (DL). Variants not affecting splicing (n=202) where divided into two groups: 89 next to a donor site and 113 next to an acceptor site.

**3.2 Cut-off selection for SpliceAI**

Firstly, the SpliceAI neural network algorithm was run for the 518 variants using the Broad Institute API (<https://spliceailookup-api.broadinstitute.org/>; accessed March 2022). It delivers four Δscores, whose interpretation is explained in the SpliceAI flagship article (Jaganathan *et al.*, 2019). A variant is considered to alter splicing when any of the Δscores exceeds the set cut-off; thus, the four Δscores were plotted simultaneously for each variant (Supplementary Figure 2). Secondly, the number of variants reaching PP3 or BP4 was calculated for each category using different cut-offs (Supplementary table 12). Since variants not affecting splicing are more frequently detected than variants affecting splicing, our dataset was biased and the results could be biased too. To avoid this, we simulated variants not affecting splicing to be nine times the 317 variants affecting splicing. This proportion would match a prior proportion of 0.1, as the one suggested in Tavtigian’s proposal for ACMG variant classification (Tavtigian *et al.*, 2018). By doing so, the final number of variants not affecting splicing was 2835, thus more weight is assigned to benign variants (Supplementary table 12).

The odds in favour of pathogenicity for every cut-off were calculated as published in Easton (2007). The cut-offs were chosen to ensure high odd ratios of pathogenicity and benignity while limiting the grey area of variants without predictive evidence.

**4. Evaluation of PS1 and PM5 criteria**

**4.1 Variant generation**

PS1 and PM5 criteria compare the novel missense change with other previously classified variants at the same amino acid residue. The previous variant must be classified using gene-specific guidelines, sometimes by the corresponding expert panel. To ensure this, the program only uses as previous variants those classified in ClinVar by Expert Panel (which for HC genes usually is ClinGen). Since our repository is downloaded from ClinGen, the comparing variants are limited. To validate the performance of PS1 and PM5, missense variants classified as pathogenic or likely pathogenic in ClinVar by Expert Panel were selected for the *ATM, CDH1, PTEN*, *TP53*, and MMR genes (<https://www.ncbi.nlm.nih.gov/clinvar/>; downloaded on May 2022).

The PS1 criterion is assigned when the novel missense variant generates the same amino acid change as a previously established pathogenic variant. From the list of missense variants, 11 new variants could be generated harbouring the same amino acid change as the original ClinGen or ClinVar Variant (Supplementary Table 14).

However, PM5 is assigned when the novel missense variant at the same codon generates a different amino acid change as a previously established pathogenic variant. There are many hypothetical variant candidates to accomplish PM5, thus, for the purpose of validation, 26 variants were chosen randomly.

**4.2 Validation**

Regarding PS1 results, 8 out of 11 variants were assigned PS1 or PS1_moderate. Three variants were denied PS1: 1) a *CDH1* variant, since PS1 do not apply to *CDH1*; 2) a *TP53* variant because the variant classified by Expert Panel was likely pathogenic and according to *TP53* guidelines only pathogenic variants should be considered; 3) a *PMS2* variant because SpliceAI prediction suggested a splicing alteration.

As for PM5, 11 of 26 variants were assigned moderate or strong strength. The cases where PM5 was denied are detailed in Supplementary table 14 and depend on gene-specific guidelines, but most were due to the criterion not being applicable to the gene, the test variant having a higher BLOSUM62 value or a higher Grantham distance score than the previous variant, or the variant having a prior Utah value score below 0.68 for MMR genes.

**Supplementary Figures**

**Supplementary Figure 1**

**List of sources queried by vaRHC. They are separeted by the type of query (local vs real-time) and the type of information ectracted from the database.**
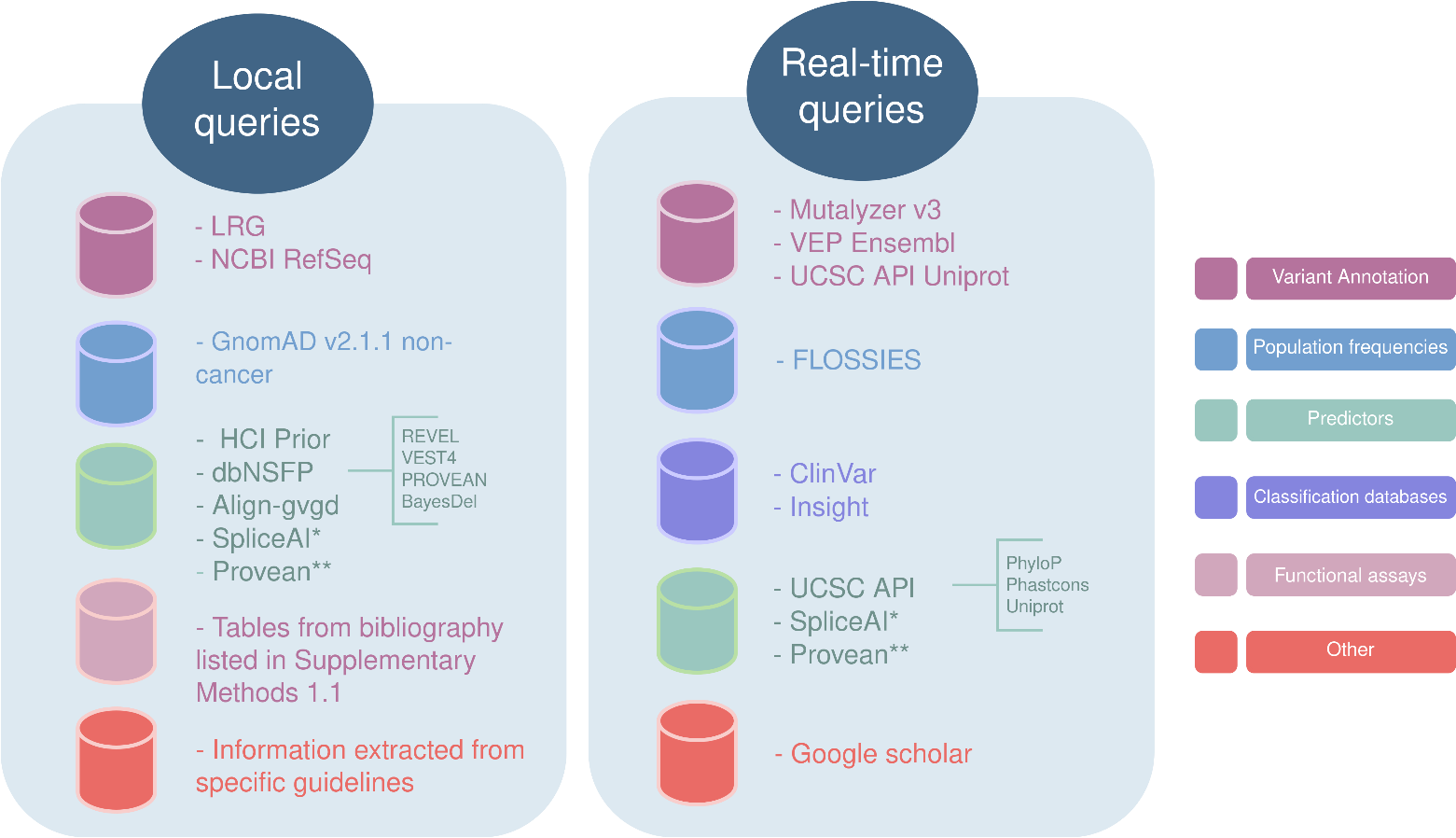


**Supplementary Figure 2**

**SpliceAI 4 Δscores plotted for each variant separated by RNA consequence.** A) Variants causing an acceptor gain; B) Variants causing a donor gain; C) Variants causing an acceptor loss; D) Variants causing a donor loss; E) Variants not altering splicing, next to an acceptor site; F) Variants not altering splicing, next to a donor site. Variants marked with colours were erroneously categorized by SpliceAI: red= altering splicing, grey= not classified, blue= not altering splicing, all according to the proposed SpliceAI thresholds.

**A)**


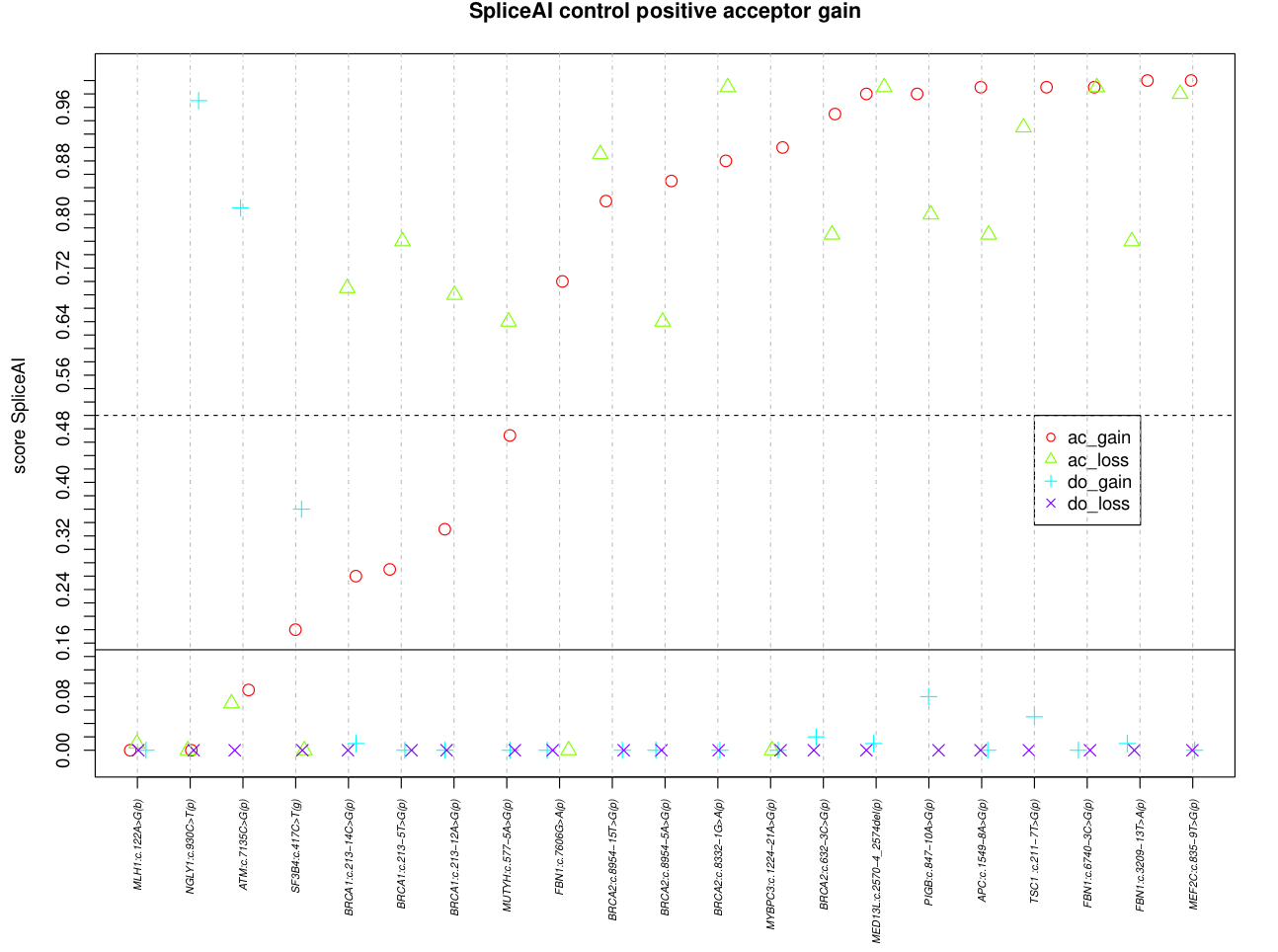

**B)**


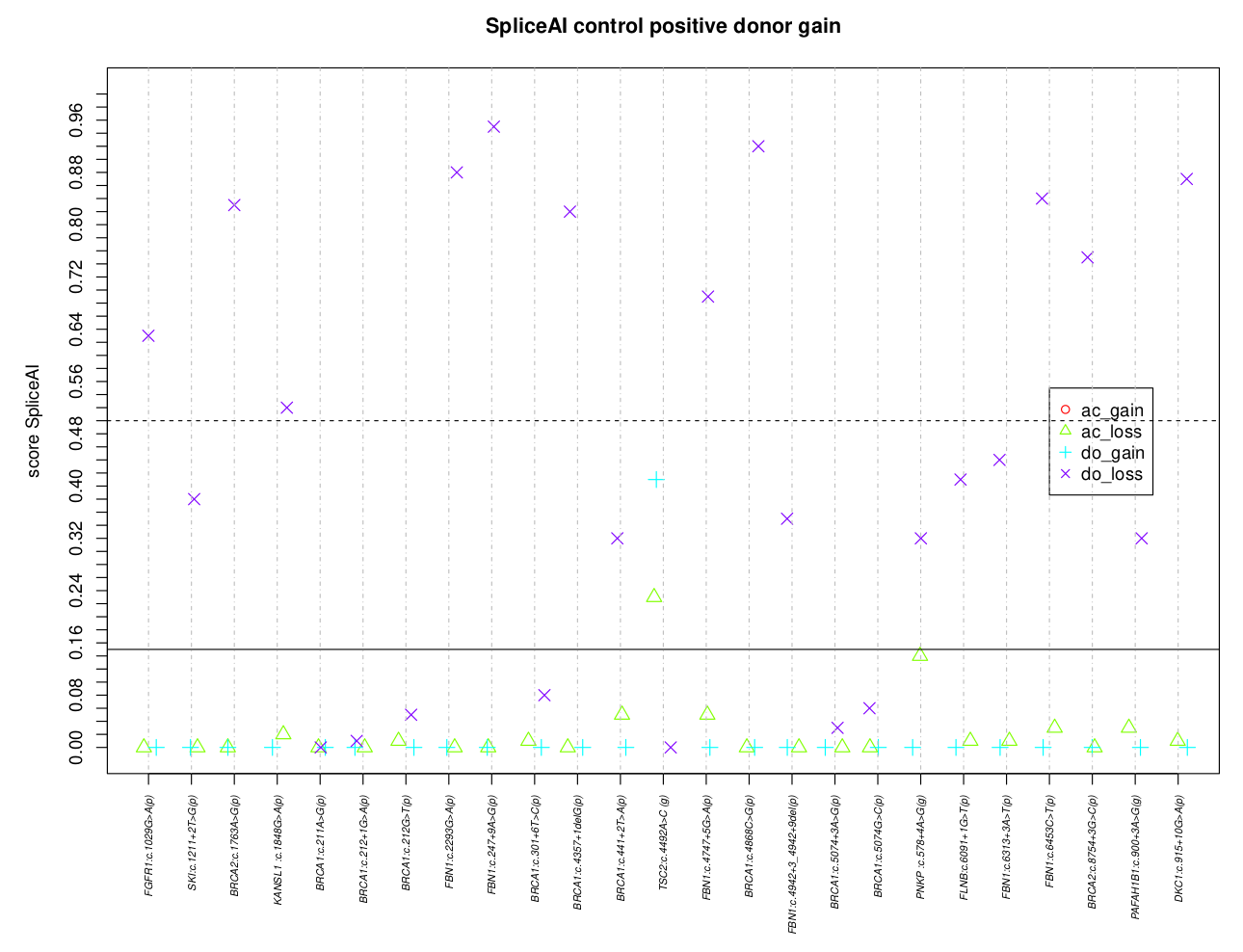

**C)**


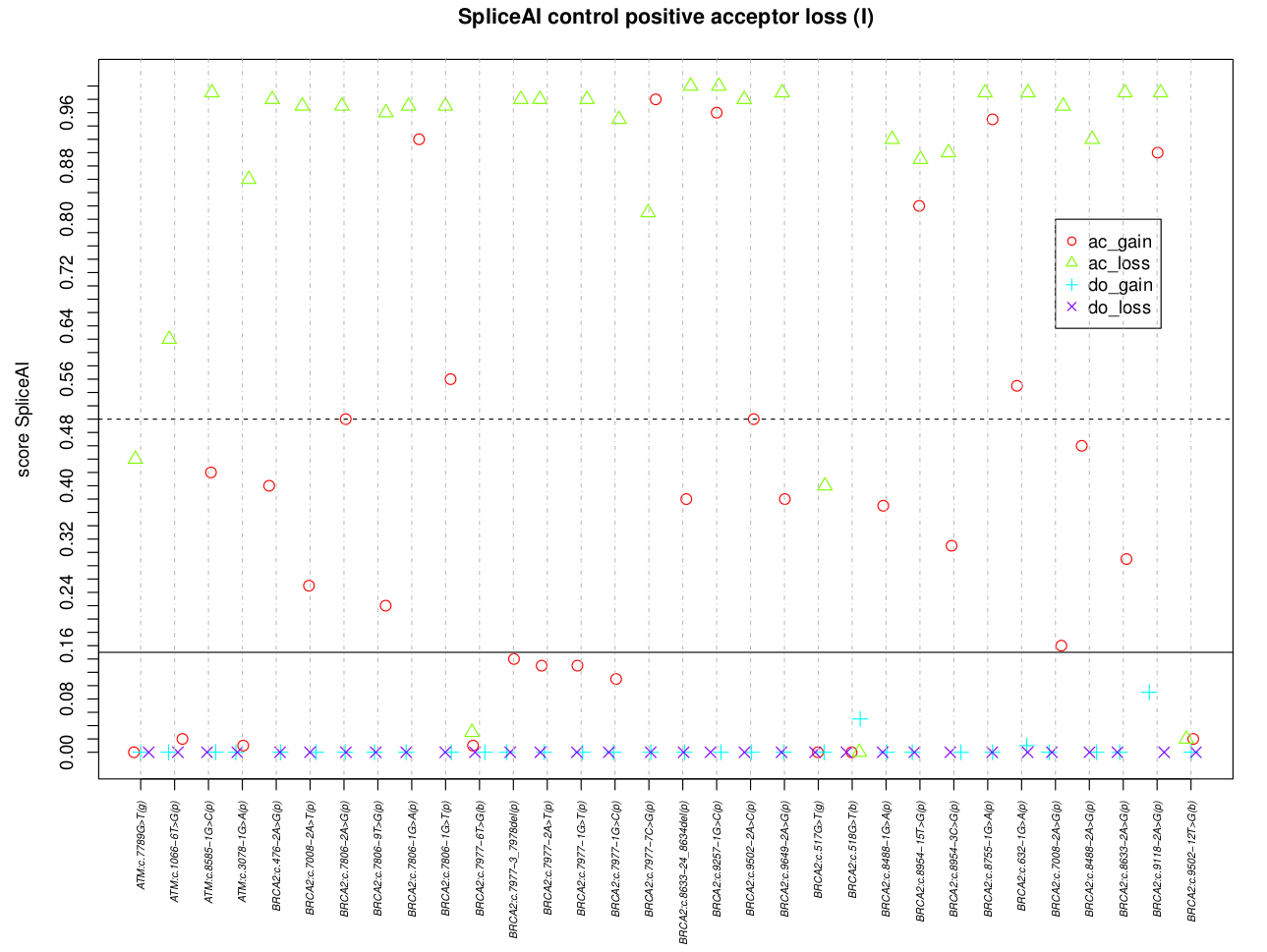

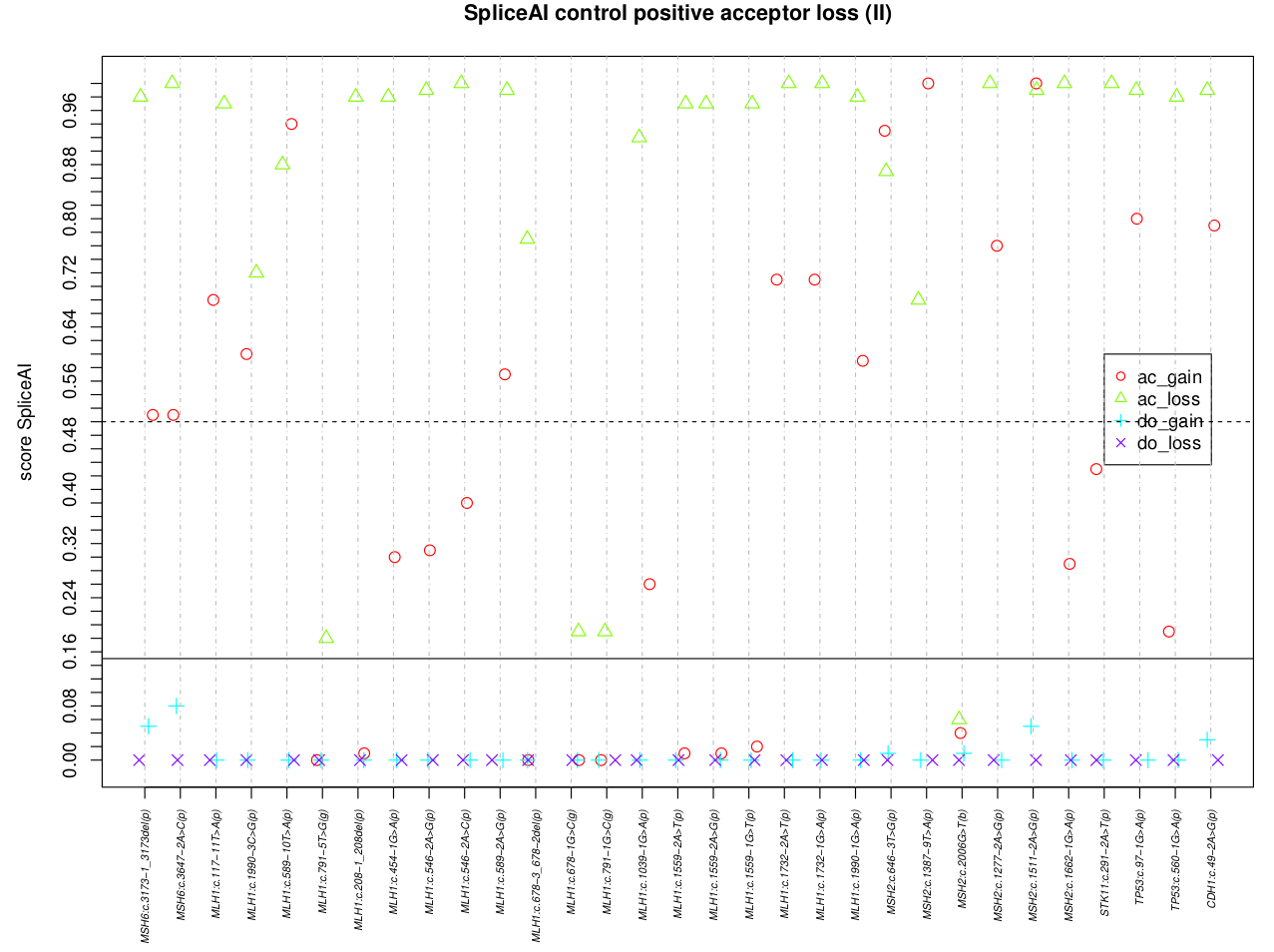

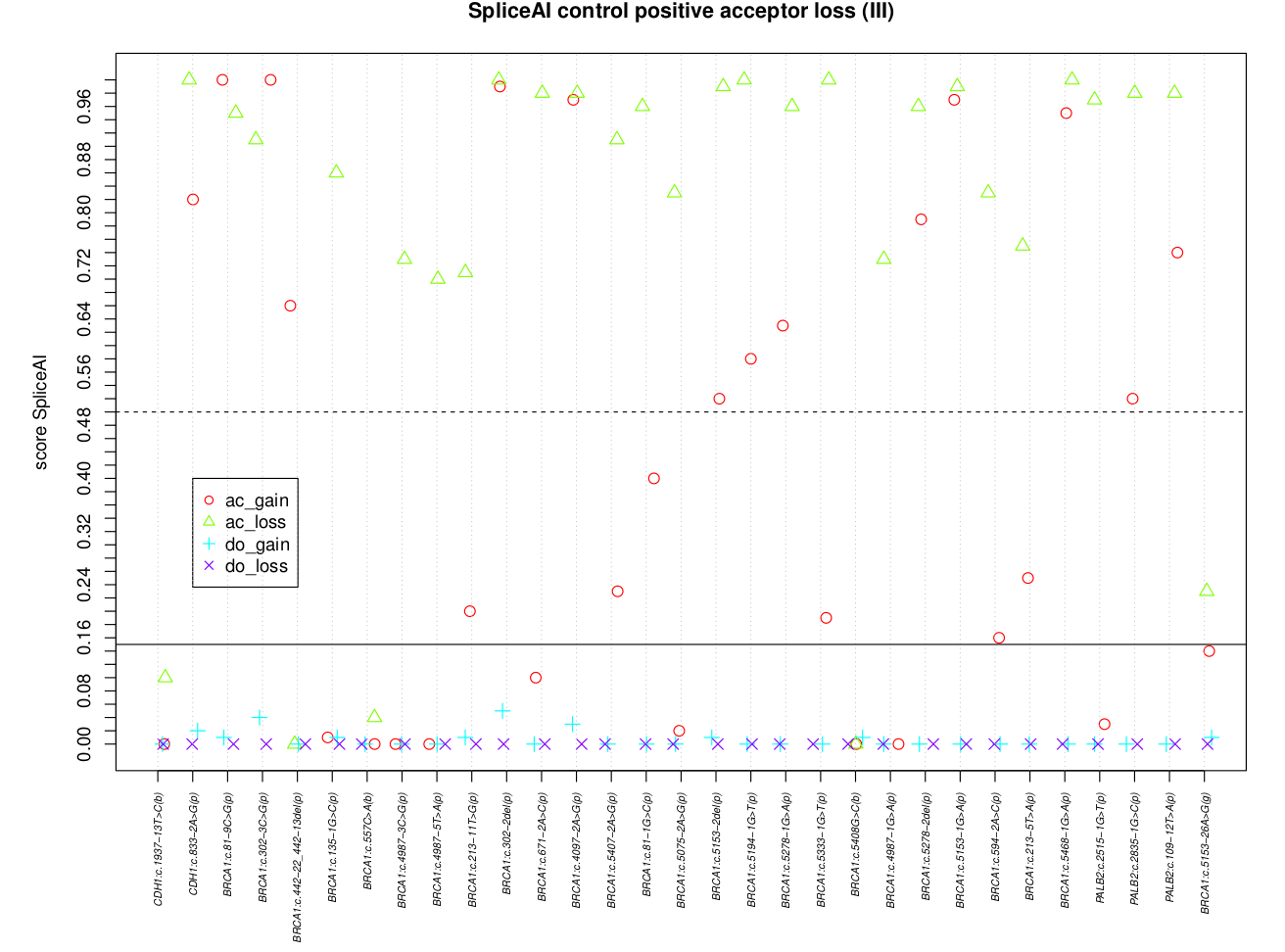

**D)**


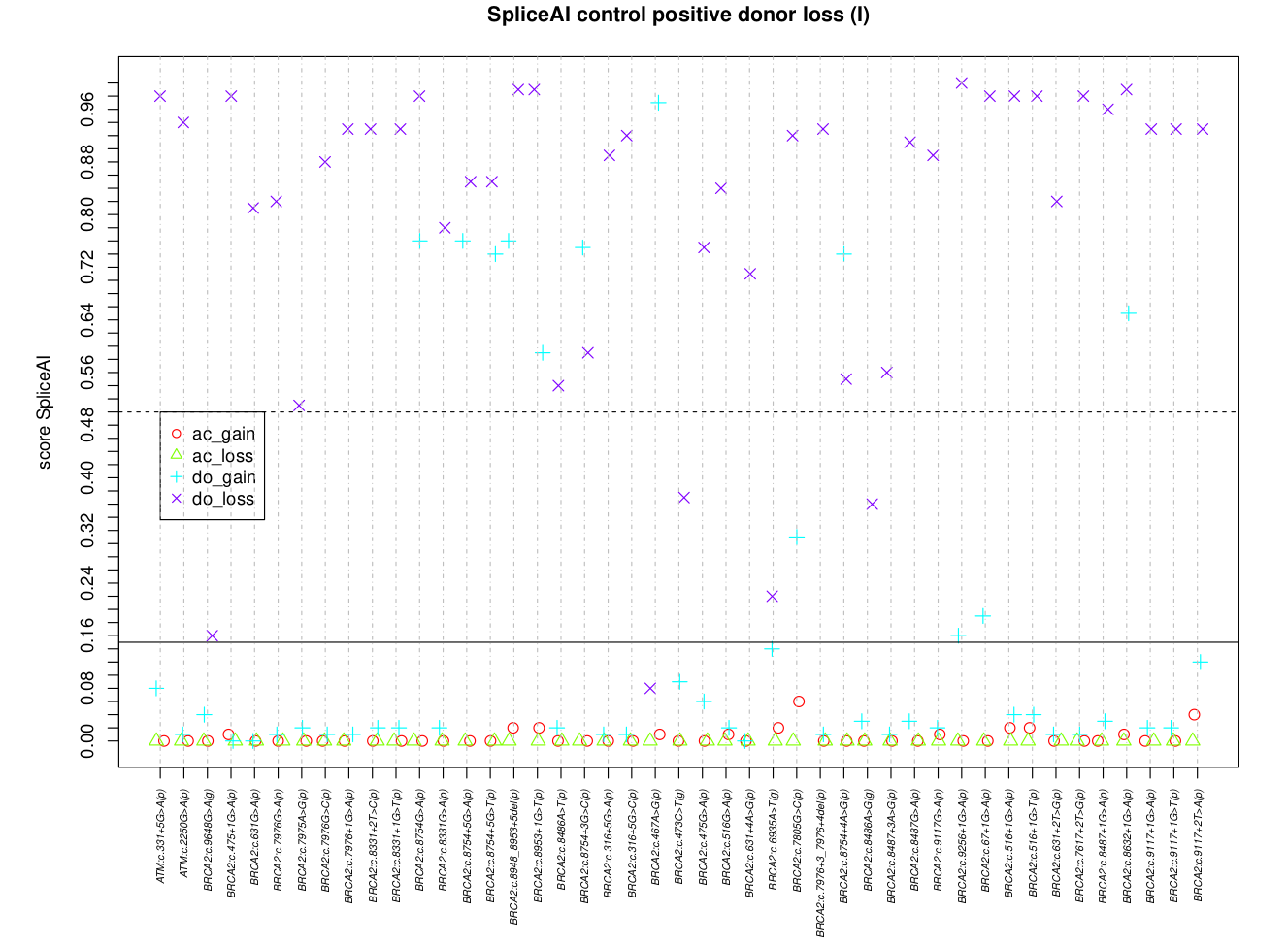

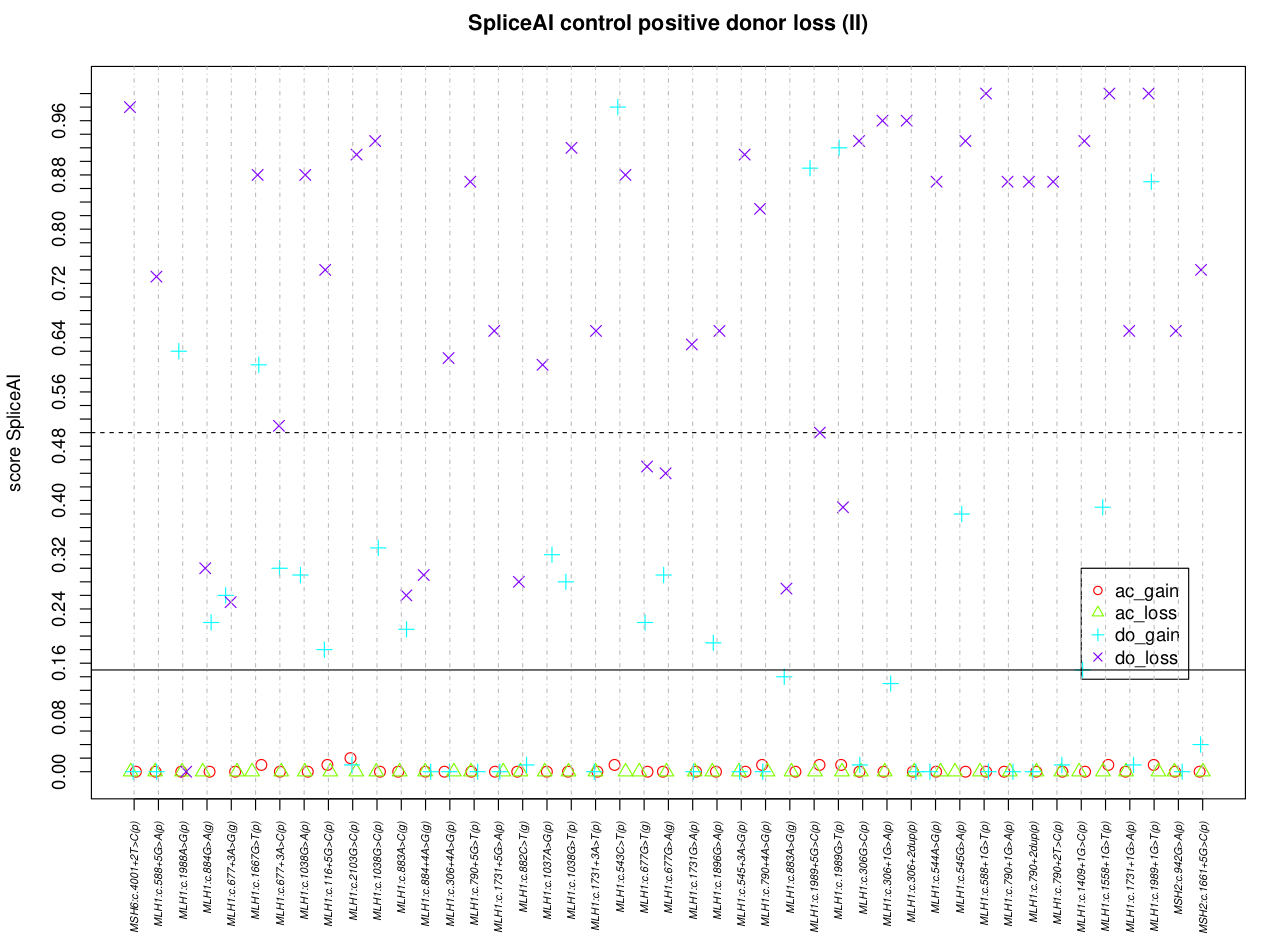

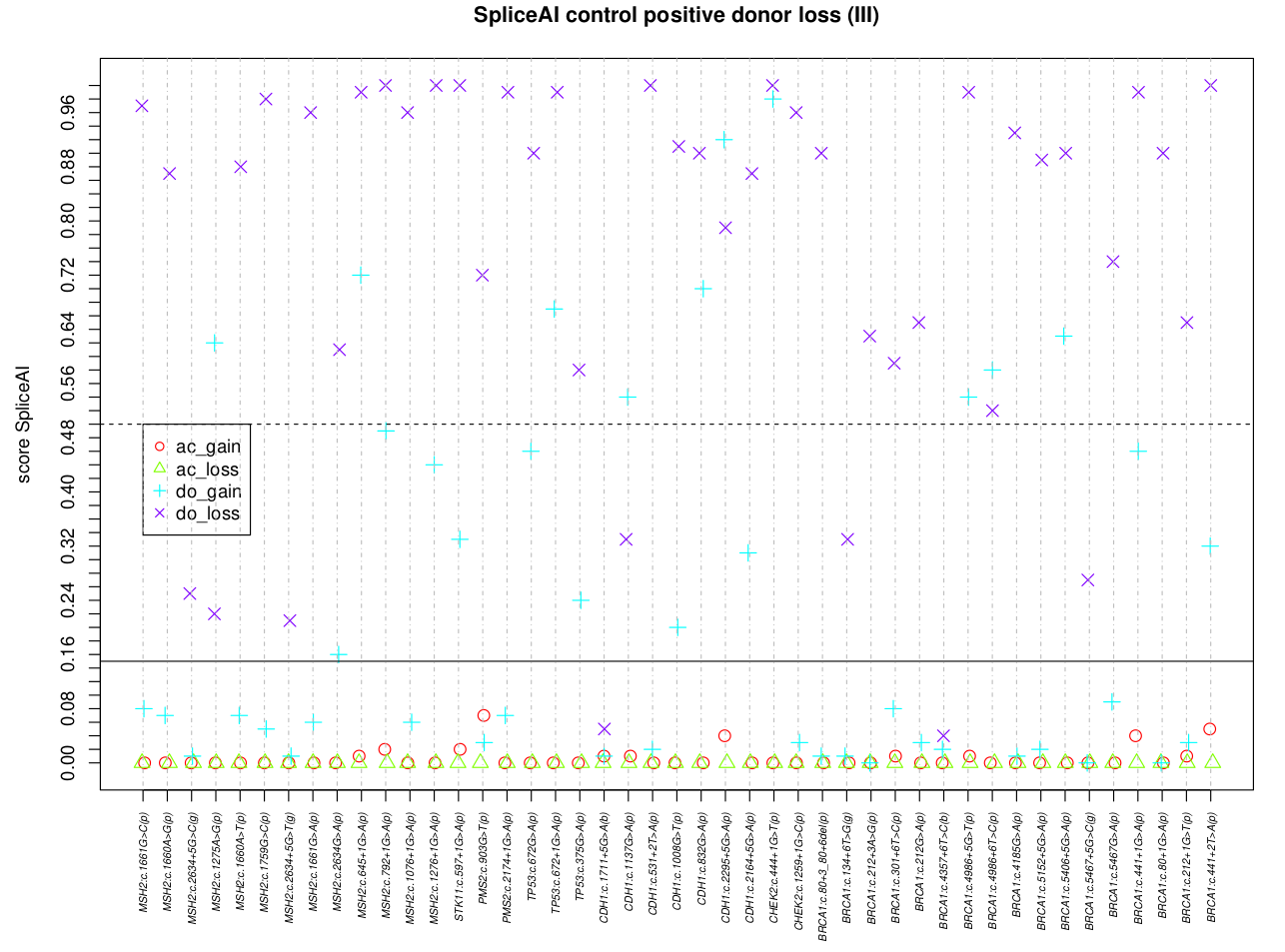

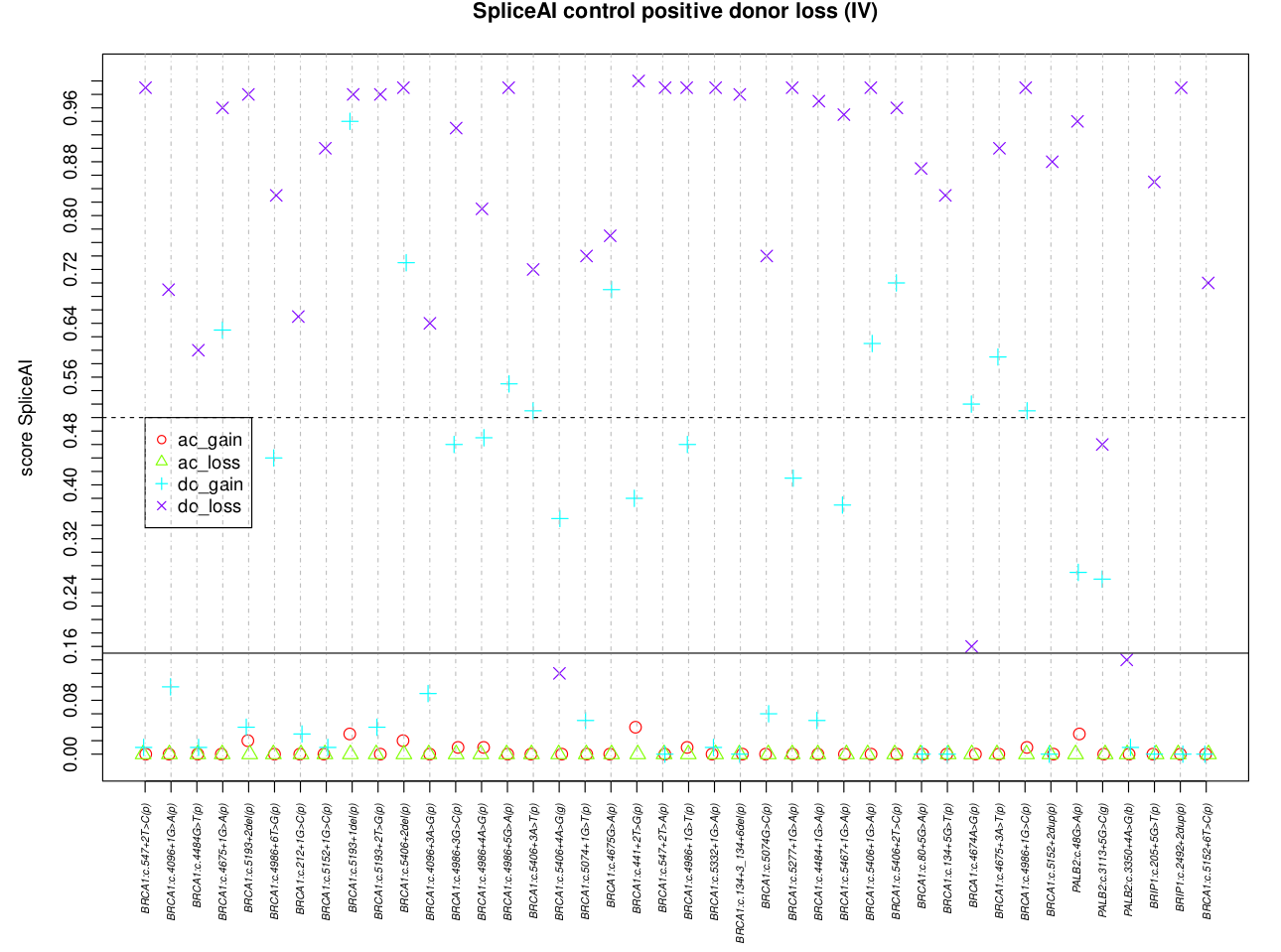

**E)**


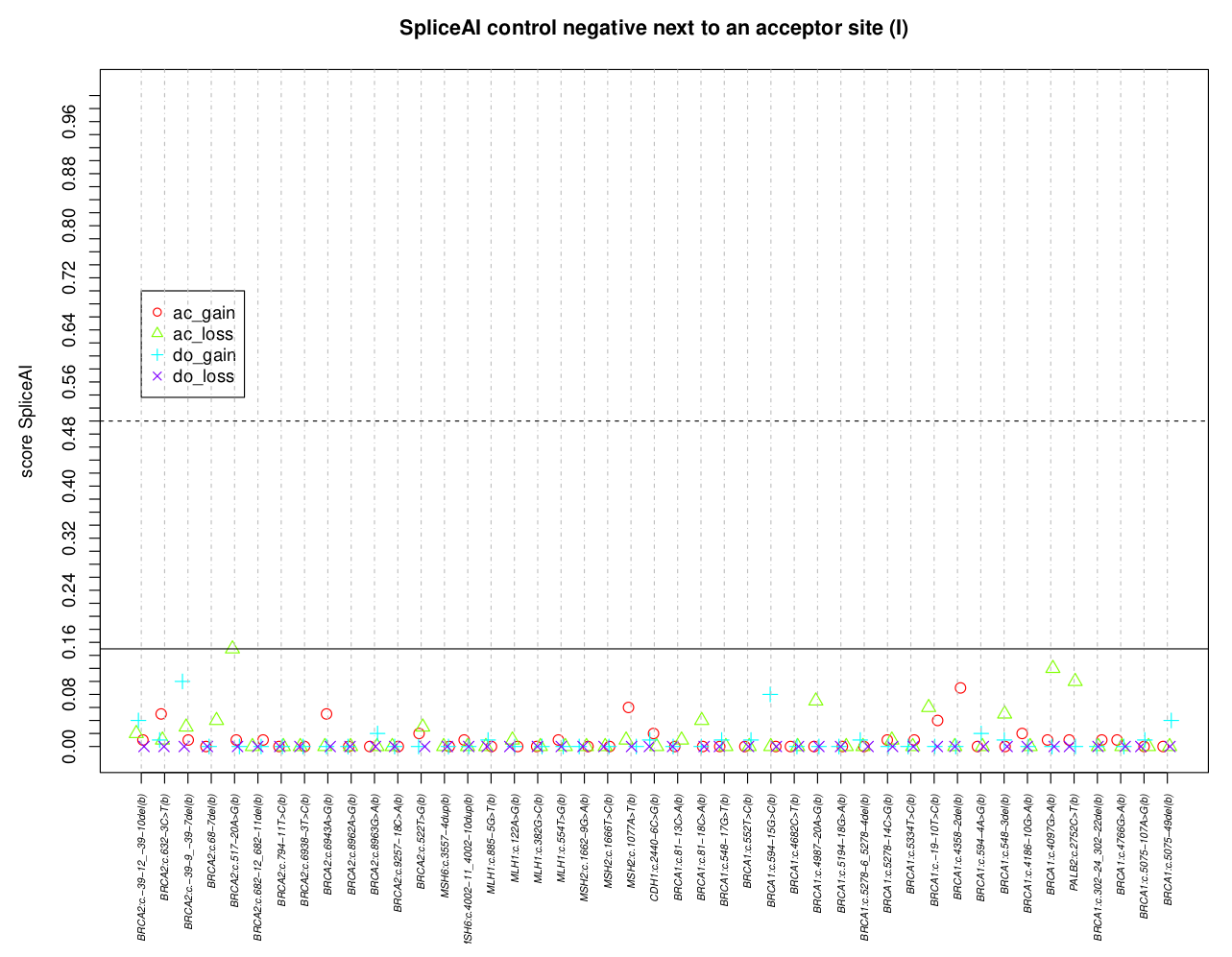


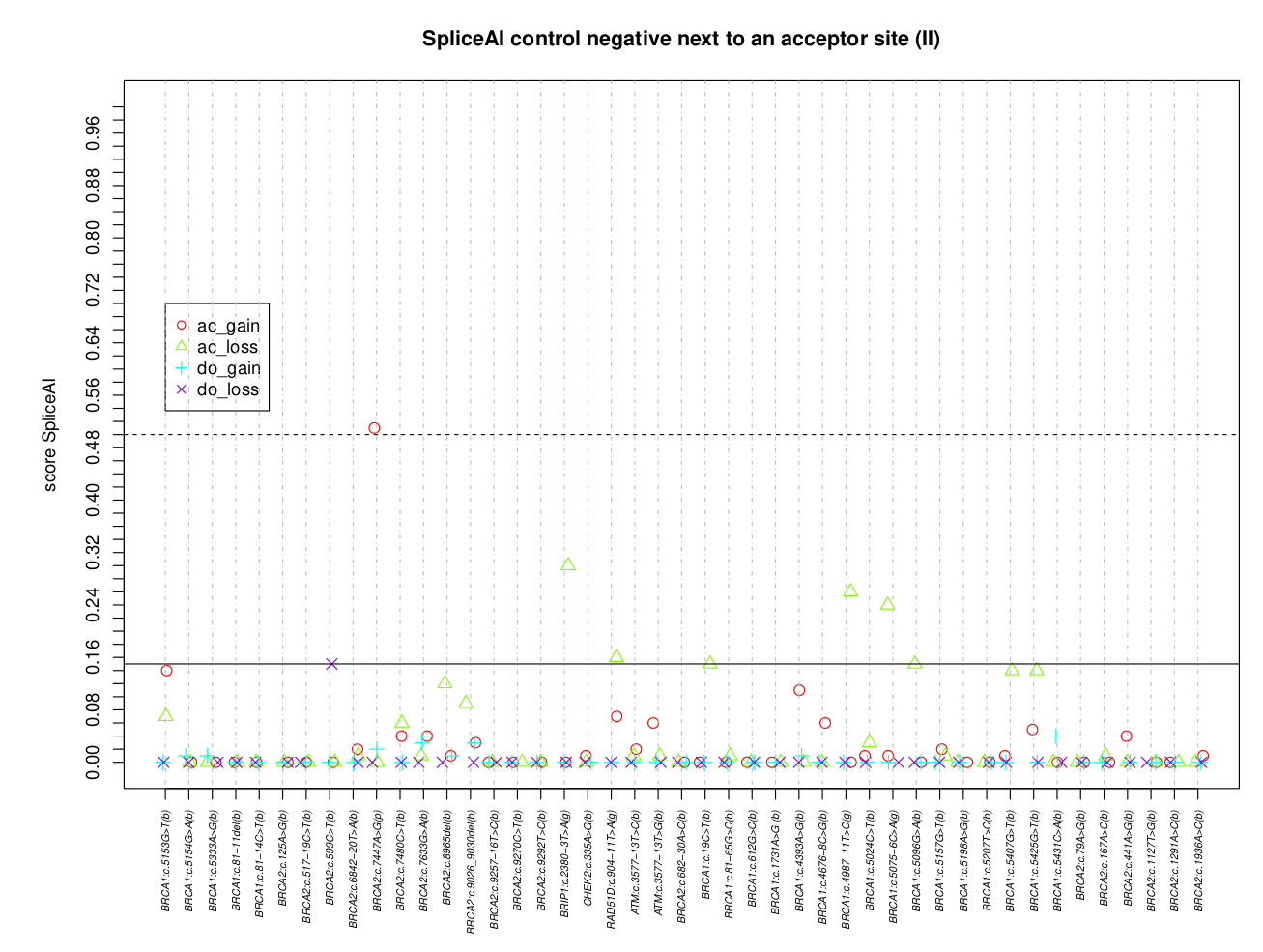

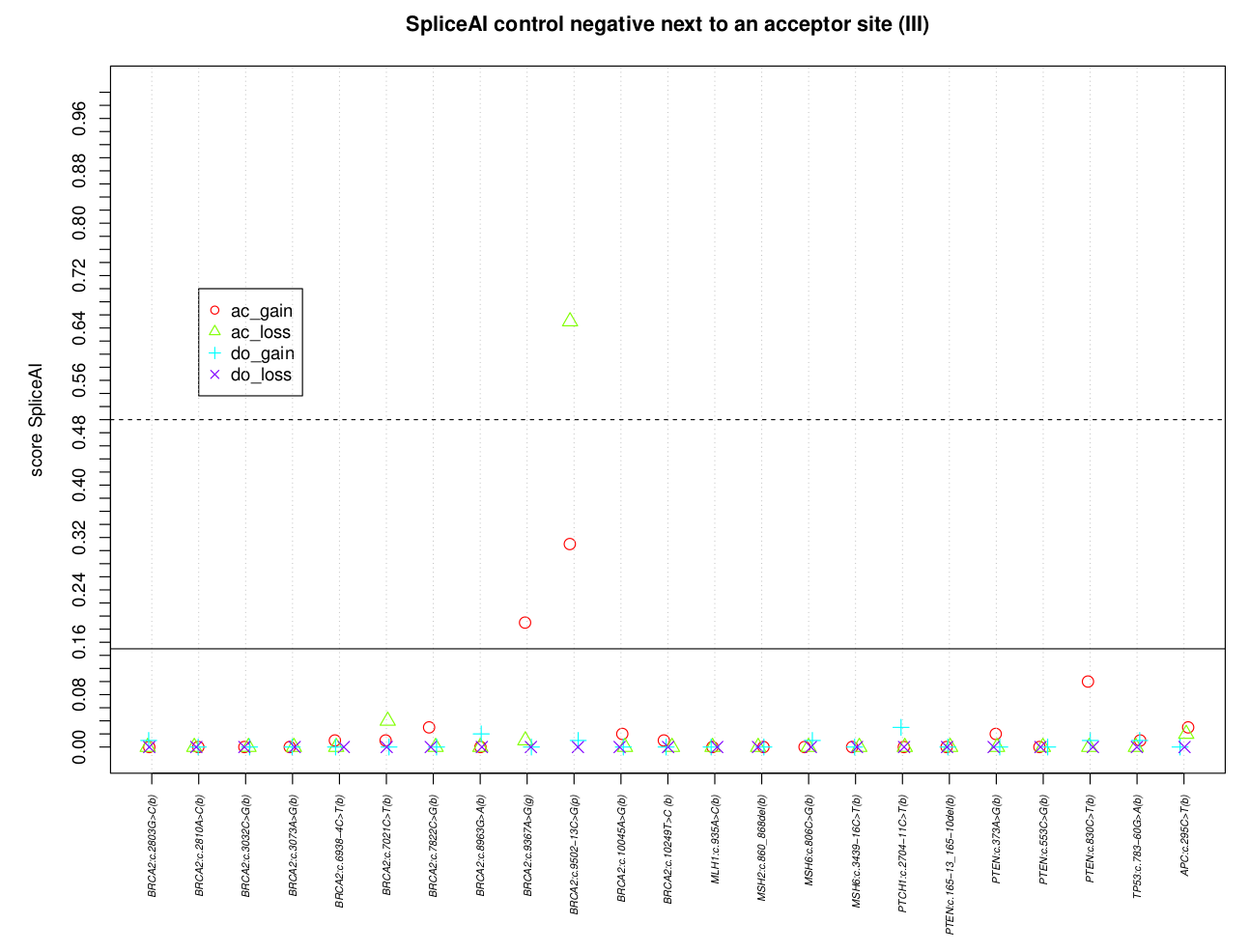

**F)**


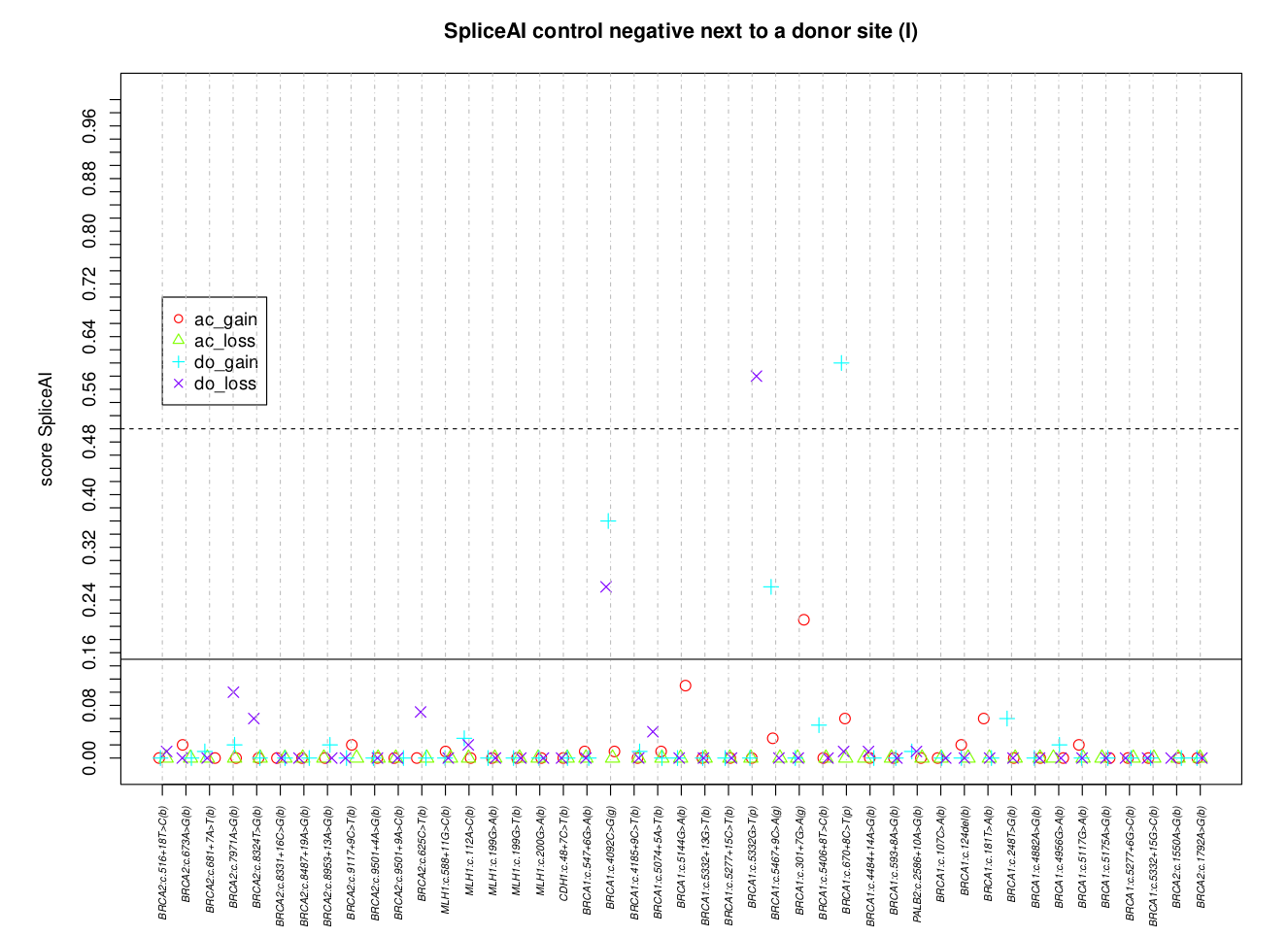

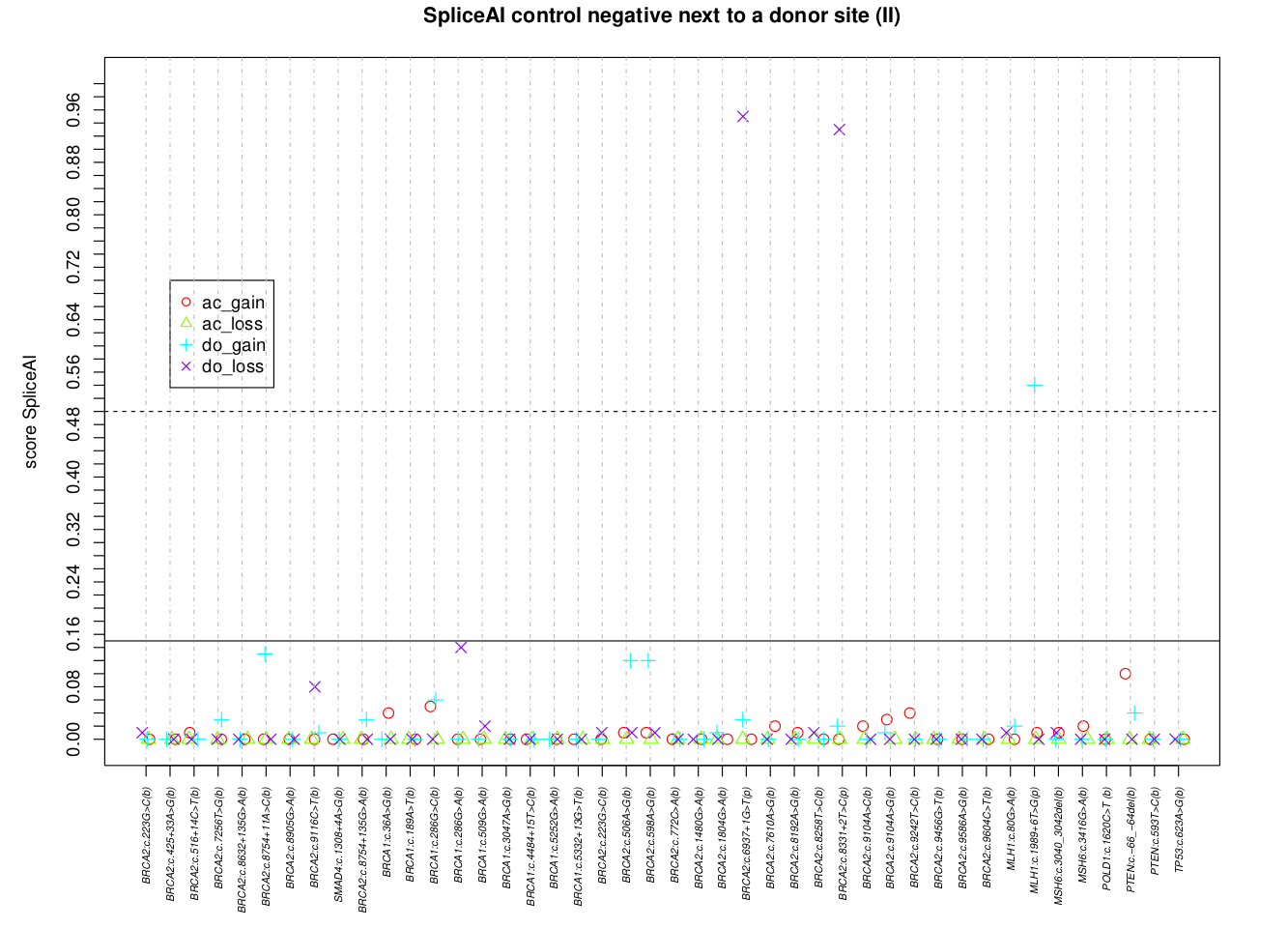

**Supplementary references**

Agrawal, S. *et al.* (2005) Different splicing defects lead to differential effects downstream of the lipid and protein phosphatase activities of PTEN. Hum. Mol. Genet., 14(16), pp. 2459–2468.

Barone, G. *et al.* (2009) Modeling ATM mutant proteins from missense changes confirms retained kinase activity. Hum. Mutat., 30(8), pp. 1222–1230.

Chen, H.J. *et al.* (2017) Characterization of cryptic splicing in germline PTEN intronic variants in Cowden syndrome. Hum. Mutat., 38(10), p. 1372.

Colombo, M. *et al.* (2014) Comprehensive annotation of splice junctions supports pervasive alternative splicing at the BRCA1 locus: a report from the ENIGMA consortium. Hum. Mol. Genet., 23(14), pp. 3666–3680.

Drost, M. *et al.* (2019) A functional assay-based procedure to classify mismatch repair gene variants in Lynch syndrome. Genet. Med., 21(7), pp. 1486–1496.

Drost, M. *et al.* (2020) Two integrated and highly predictive functional analysis-based procedures for the classification of MSH6 variants in Lynch syndrome. Genet. Med. 2020 225, 22(5), pp. 847–856.

Easton, D.F. *et al.* (2007) A systematic genetic assessment of 1,433 sequence variants of unknown clinical significance in the BRCA1 and BRCA2 breast cancer-predisposition genes. Am. J. Hum. Genet., 81(5), pp. 873–883.

Giacomelli, A.O. *et al.* (2018) Mutational processes shape the landscape of TP53 mutations in human cancer. Nat. Genet., 50(10), pp. 1381–1387.

*InSiGHT variants databases - InSiGHT* (2022).

Jaganathan, K. *et al.* (2019) Predicting Splicing from Primary Sequence with Deep Learning. Cell, 176(3), pp. 535-548.e24.

Jia, X. *et al.* (2021) Massively parallel functional testing of MSH2 missense variants conferring Lynch syndrome risk. Am. J. Hum. Genet., 108(1), pp. 163–175.

Karczewski, K.J. *et al.* (2020) The mutational constraint spectrum quantified from variation in 141,456 humans. Nat. 2020 5817809, 581(7809), pp. 434–443.

Kato, S. *et al.* (2003) Understanding the function-structure and function-mutation relationships of p53 tumor suppressor protein by high-resolution missense mutation analysis. Proc. Natl. Acad. Sci. U. S. A., 100(14), pp. 8424–8429.

Kotler, E. *et al.* (2018) Functional characterization of the p53 ‘mutome’. Mol. Cell. Oncol., 5(6).

Landrum, M.J. *et al.* (2014) ClinVar: public archive of relationships among sequence variation and human phenotype. Nucleic Acids Res., 42(D1), pp. D980–D985.

Landrum, M.J. *et al.* (2018) ClinVar: improving access to variant interpretations and supporting evidence. Nucleic Acids Res., 46(D1), pp. D1062–D1067.

Lefter, M. *et al.* (2021) Next Generation HGVS Nomenclature Checker. Bioinformatics, 37(18), pp. 2811–2817.

Liu, X. *et al.* (2020) dbNSFP v4: a comprehensive database of transcript-specific functional predictions and annotations for human nonsynonymous and splice-site SNVs. Genome Med., 12(1), pp. 1–8.

Lyra, P.C.M. *et al.* (2020) Integration of functional assay data results provides strong evidence for classification of hundreds of BRCA1 variants of uncertain significance. Genet. Med. 2020 232, 23(2), pp. 306–315.

McLaren, W. *et al.* (2016) The Ensembl Variant Effect Predictor. Genome Biol., 17(1), pp. 1–14.

Menéndez, M. *et al.* (2012) Assessing the RNA effect of 26 DNA variants in the BRCA1 and BRCA2 genes. Breast Cancer Res. Treat., 132(3), pp. 979–992.

Mitui, M. *et al.* (2009) Functional and computational assessment of missense variants in the ataxia-telangiectasia mutated (ATM) gene: mutations with increased cancer risk. Hum. Mutat., 30(1), pp. 12–21.

Parsons, M.T. *et al.* (2019) Large scale multifactorial likelihood quantitative analysis of BRCA1 and BRCA2 variants: An ENIGMA resource to support clinical variant classification. Hum. Mutat., 40(9), pp. 1557–1578.

Quiles, F. *et al.* (2016) Investigating the effect of 28 BRCA1 and BRCA2 mutations on their related transcribed mRNA. Breast Cancer Res. Treat., 155(2), pp. 253–260.

Rayner, E. *et al.* (2022) Predictive functional assay-based classification of PMS2 variants in Lynch syndrome. Hum. Mutat. [Preprint].

Rofes, P. *et al.* (2020) Improving Genetic Testing in Hereditary Cancer by RNA Analysis: Tools to Prioritize Splicing Studies and Challenges in Applying American College of Medical Genetics and Genomics Guidelines. J. Mol. Diagn., 22(12), pp. 1453–1468.

Schwarz, J.K. *et al.* (2003) Regulation of the Chk2 Protein Kinase by Oligomerization-Mediated cis-and trans-Phosphorylation.

Scott, S.P. *et al.* (2002) Missense mutations but not allelic variants alter the function of ATM by dominant interference in patients with breast cancer. Proc. Natl. Acad. Sci. U. S. A., 99(2), pp. 925–930.

Sodha, N. *et al.* (2006) Rare Germ Line CHEK2 Variants Identified in Breast Cancer Families Encode Proteins That Show Impaired Activation. Cancer Res., 66(18), pp. 8966–8970.

Thomassen, M. *et al.* (2012) Characterization of BRCA1 and BRCA2 splicing variants: a collaborative report by ENIGMA consortium members. Breast Cancer Res. Treat., 132(3), pp. 1009–1023.

Trojan, J. *et al.* (2001) Activation of a cryptic splice site of PTEN and loss of heterozygosity in benign skin lesions in Cowden disease. J. Invest. Dermatol., 117(6), pp. 1650–1653.

Whiley, P.J. *et al.* (2014) Comparison of mRNA splicing assay protocols across multiple laboratories: recommendations for best practice in standardized clinical testing. Clin. Chem., 60(2), pp. 341–352.

Wu, X. *et al.* (2006) Characterization of CHEK2 mutations in prostate cancer. Hum. Mutat., 27(8), pp. 742–747.

Wu, X. *et al.* (2001) Characterization of tumor-associated Chk2 mutations. J. Biol. Chem., 276(4), pp. 2971–2974.
